# Competitive and Cooperative Interactions Mediate RNA Transfer from Herpesvirus Saimiri ORF57 to the Mammalian Export Adaptor ALYREF

**DOI:** 10.1371/journal.ppat.1003907

**Published:** 2014-02-13

**Authors:** Richard B. Tunnicliffe, Guillaume M. Hautbergue, Stuart A. Wilson, Priti Kalra, Alexander P. Golovanov

**Affiliations:** 1 Manchester Institute of Biotechnology and Faculty of Life Sciences, The University of Manchester, Manchester, United Kingdom; 2 RNA Biology Laboratory, Sheffield Institute for Translational Neuroscience, Department of Neuroscience, University of Sheffield, Sheffield, United Kingdom; 3 Department of Molecular Biology and Biotechnology, University of Sheffield, Sheffield, United Kingdom; University of Utah, United States of America

## Abstract

The essential herpesvirus adaptor protein HVS ORF57, which has homologs in all other herpesviruses, promotes viral mRNA export by utilizing the cellular mRNA export machinery. ORF57 protein specifically recognizes viral mRNA transcripts, and binds to proteins of the cellular transcription-export (TREX) complex, in particular ALYREF. This interaction introduces viral mRNA to the NXF1 pathway, subsequently directing it to the nuclear pore for export to the cytoplasm. Here we have used a range of techniques to reveal the sites for direct contact between RNA and ORF57 in the absence and presence of ALYREF. A binding site within ORF57 was characterized which recognizes specific viral mRNA motifs. When ALYREF is present, part of this ORF57 RNA binding site, composed of an α-helix, binds preferentially to ALYREF. This competitively displaces viral RNA from the α-helix, but contact with RNA is still maintained by a flanking region. At the same time, the flexible N-terminal domain of ALYREF comes into contact with the viral RNA, which becomes engaged in an extensive network of synergistic interactions with both ALYREF and ORF57. Transfer of RNA to ALYREF in the ternary complex, and involvement of individual ORF57 residues in RNA recognition, were confirmed by UV cross-linking and mutagenesis. The atomic-resolution structure of the ORF57-ALYREF interface was determined, which noticeably differed from the homologous ICP27-ALYREF structure. Together, the data provides the first site-specific description of how viral mRNA is locked by a herpes viral adaptor protein in complex with cellular ALYREF, giving herpesvirus access to the cellular mRNA export machinery. The NMR strategy used may be more generally applicable to the study of fuzzy protein-protein-RNA complexes which involve flexible polypeptide regions.

## Author Summary

Herpes viruses invade cells, hijacking cellular components to sustain their lifecycle and replicate. A critical step of infection is the export of viral mRNA from the nucleus to the cytoplasm, where the molecular machinery to produce proteins is located. To provide a link between their mRNA and cellular components of the mRNA export pathway, all herpesviruses use special adaptor proteins. These adaptor proteins specifically select viral mRNAs from the mixture present in the nucleus, and introduce them to cellular mRNA export factors, such as ALYREF. How these viral adaptors manage to trick ALYREF to accept foreign genetic material has not been understood on a molecular level. In this study we reveal how a typical viral adaptor protein ORF57 recognizes specific viral RNA motifs, and also how it binds to the cellular protein ALYREF. We uncover details of how ORF57 transfers the viral RNA to ALYREF, locking it in the cooperative ternary complex. We also describe the atomic-resolution structure of ORF57-ALYREF interaction interface. Together the data provides the first molecular insight of how viral mRNA is transferred between viral and cellular proteins, thus helping virus to hijack a cell.

## Introduction

Mammalian gene expression is coupled with mRNA maturation, where nascent transcripts undergo a continuous series of splicing and processing events finally leading to nuclear export to the cytoplasm [1]. This process is tightly regulated and orchestrated, ensuring that only mature and fully-processed cellular mRNA is exported from the nucleus, to be correctly translated into proteins in the cytoplasm. The recruitment of protein markers acquired during this maturation process, such as UAP56, UIF and ALYREF (otherwise known as Aly, REF, Aly/REF, REF/Aly, BEF, Thoc4 in metazoan and Yra1 in yeast), is essential for the export of cellular mRNA via the NXF1 pathway (otherwise known as TAP) [2]–[4]. These markers are part of the multicomponent TREX complex which associates with the 5′ end of cellular mRNAs during splicing [3]. TREX recruits NXF1 to mRNA and TREX triggers a conformational change in NXF1, such that it binds mRNA with high affinity [5], [6]. The cellular protein ALYREF functions as an export adaptor, binding mRNA as part of TREX, and also interacting with NXF1 [7], [8]. The structure of ALYREF has been characterized: it consists of central folded RRM domain [9] flanked by two largely flexible multifunctional N- and C-terminal domains [10]. ALYREF primarily uses its N-terminal flexible arginine-rich region for interaction with NXF1; this region closely overlaps with the RNA binding site [10]. The arginines within this region become methylated, which reduces its RNA binding activity and may serve as a control mechanism for RNA displacement from ALYREF to NXF1 [11]. ALYREF and Thoc5 binding remodels NXF1, increasing its binding affinity for mRNA, ensuring transfer of mRNA to NXF1 [5], [6]. NXF1 then introduces the mRNA to nucleoporins, committing it to exit from the nucleus through the nuclear pore [12]–[14].

Herpesviridae possess an intriguing ability to circumvent the sophisticated cellular controls which ensure that only mature spliced mRNA can be exported from the nucleus. Viral mRNA is generally unspliced, therefore it cannot acquire the normal protein markers during splicing, which would signal that mRNA is ready for export to the cytoplasm. However, all herpesviruses express an essential multi-functional adaptor protein which specifically recognizes viral mRNA, and bridges its interaction with TREX complex via binding to cellular mRNA export factors such as ALYREF and UIF [15]–[19], for subsequent export via the NXF1 pathway [20]–[23]. It was also recently suggested that ALYREF may be recruited by viral adaptors to stabilize the viral nuclear RNAs independently of their export [24]. The infected cell protein 27 (ICP27) from Herpes Simplex Virus type 1 (HSV-1) is probably one of the most well-studied examples of the viral multifunctional adaptors [25]. In Herpesvirus Saimiri (HVS), which is the prototype γ-2 herpesvirus with close similarity to human Kaposi's Sarcoma-associated herpesvirus (KSHV), a similar function is carried out by the protein ORF57 [21], [23]. Homologs of these adaptor proteins are also known as ORF57 in KSHV [26], [27], EB2 in Epstein-Barr virus (EBV) [28], and UL69 in the human cytomegalovirus [29]. All these viral adaptor proteins contain long intrinsically-unstructured but functionally-important regions, with relatively poor sequence homology. Although these proteins appear to have a very similar function in promoting viral mRNA export via the cellular NXF1 pathway, the location and appearance of their RNA-binding regions vary, and the precise location of ALYREF binding sites cannot be inferred from their amino acid sequences. How exactly they perform their viral mRNA export function, and introduce viral mRNA to cellular proteins such as ALYREF, has not been described in detail yet.

Recently, the structure of the interaction interface between HSV-1 adaptor protein ICP27 and cellular mRNA export factor ALYREF was determined [30]. (It should be noted that while in our previous study [30] ALYREF protein was referred to as REF, due to recent recommended changes by the HUGO Gene Nomenclature Committee [31] here we will be referring to the same protein as ALYREF). In this structure, interaction with the RRM domain of ALYREF is achieved via a very short peptide fragment of the flexible N-terminal region of ICP27 [30]. Additionally, a ALYREF-interacting region aa103–120 was mapped on HVS ORF57 protein [30]. The mostly unstructured ORF57 region aa8–120 [30] mediates specific recognition of HVS mRNA via the viral RNA sequence motif GAAGRG [32]. Although this ORF57 fragment contains an arginine-rich region, it lacks any canonical RNA-binding sequence features such as an RGG box, which is present in ICP27 [33], [34]. Therefore, the exact location of the RNA binding site remained unknown, along with the mechanism of RNA transfer from ORF57 to ALYREF. Which protein sites are involved at different stages of such a transfer? What is the structure of the ternary ORF57-RNA-ALYREF complex? Answers to these questions would enable further functional and mutagenesis studies, to reveal how the assembly and disassembly of complexes involved in RNA recognition, transfer and export are achieved at the molecular level [35], [36].

In this study we used solution state NMR to reveal molecular details of the ternary complex assembly of functional fragments of HVS ORF57, HVS RNA and ALYREF, and suggest a model for the mechanism of RNA transfer between protein molecules in this system. The mapping experiments show a clear difference between binding of non-specific random-sequence RNA oligos, and RNA oligos containing HVS-specific sequence motifs, to a flexible arginine-rich region of ORF57. We reveal that for the ORF57 protein, its ALYREF binding site also forms part of the specific viral RNA recognition region, with adjacent arginine-rich sequences also contributing to RNA binding. We present the atomic-resolution structure of the ORF57-ALYREF binding interface, which somewhat differs from that of ICP27-ALYREF identified earlier [30]. Using a new strategy based on principles of saturation-transfer (ST) between molecules [37] and isotopically-discriminated NMR [38], we followed the changes in RNA binding sites which accompany transfer of RNA from one protein molecule to another. In the ternary ORF57-RNA-ALYREF complex, RNA is partially displaced from its binding site on ORF57 by ALYREF, but is retained in the complex by the synergistic action of flanking flexible regions of both ALYREF and ORF57. The detailed model obtained based on NMR data was supported by mutagenesis studies, and cooperativity in ternary complex assembly was additionally characterized by fluorescence measurements.

## Results

### Mapping the specific *HVS* mRNA binding site on ORF57

**Figure 1:**
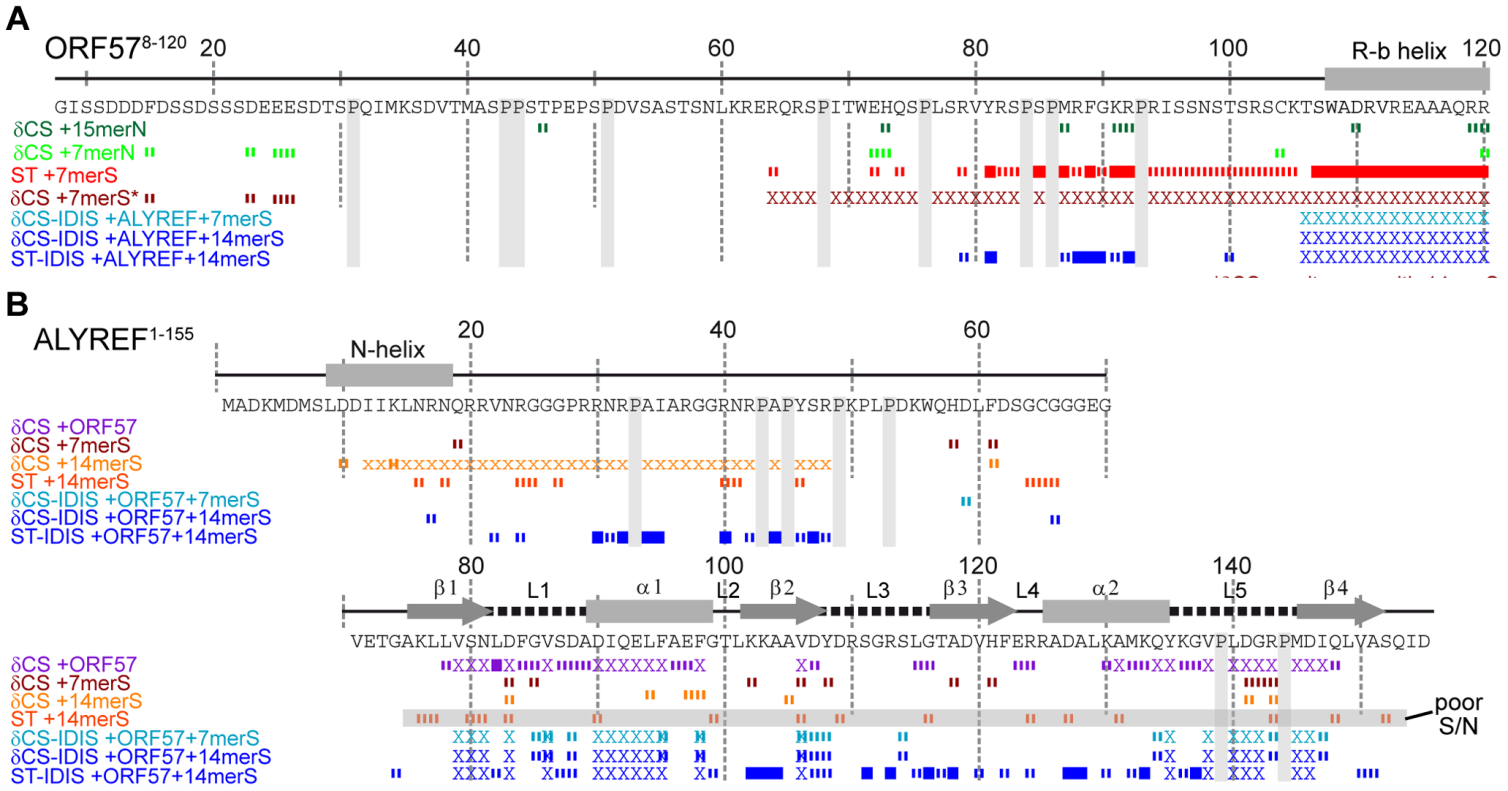
Overview of RNA interaction sites mapped on ORF57^8–120^ and ALYREF^1–155^ using different approaches. Backbone amide chemical shift change (δCS) and saturation transfer (ST) data are shown for ORF57^8–120^ (A) and ALYREF^1–155^ (B) with different ligands added as labeled. Crosses indicate residues with signal broadened beyond detection. For δCS, large (>0.1) and moderate (>0.04) chemical shift changes of each protein relative to signals in its free state, are shown as solid and broken bars, respectively. For ST data, signal intensity ratios significantly different from the background mean values are represented by solid (>6 SD) and broken (>3 SD) bars, respectively. Labels indicate the state of the protein for each dataset, data shown for interactions with non-specific RNA (green), ORF57 specific RNA (red/orange) and ALYREF-ORF57-RNA complex (blue).

Previously the recognition of specific short viral mRNA sequences was attributed to Herpesvirus saimiri ORF57 protein region aa8–120 [32], however the precise binding site within this fairly long region was unknown, and no obvious sequence patterns (such as RGG boxes) indicative of RNA-binding sites could be identified. To locate the RNA-binding regions experimentally within ORF57^8–120^, and study specific vs non-specific binding, we used NMR spectroscopy. Sequence specific signal assignments of all amides of ORF57^8–120^ allowed the mapping of interaction sites to a residue-level resolution. The unlabelled RNA oligonucleotides were added to ^15^N-labelled protein samples and residue-specific signal changes were monitored. The effect of non-specific RNAs of different lengths (oligonucleotides named 7merN and 15merN, see Materials section) on signal position and shape were compared with ORF57-specific RNA oligos (7merS and 14merS) containing the previously identified HVS motif GAAGAG [32] (Fig. 1A and Fig. S1A). For non-specific 7merN and 15merN (even at two-fold excess) the amide signal changes in ORF57^8–120^ were small and scattered across the entire sequence (Fig. S1A), suggesting only transient non-specific binding. Similarly, addition of a two-fold excess of non-specific 7merN caused no significant change in local mobility of the ORF57 polypeptide chain, as evidenced by ^15^N{^1^H}-NOE both for the ORF57^8–120^ and ORF57^56–140^ constructs (Fig. S1B). (The latter construct was used as a control to ensure that the absence of binding with 7merN is not an artifact of C-terminal truncation of a potential binding site). In contrast, the ORF57-specific oligos caused substantial signal broadening in all signals corresponding to the region aa64–120 (Fig. 1A). The severity of signal perturbations was also dramatically dependent on the length of the oligo used, reflecting differences in the apparent affinity of RNA binding. For specific 7merS, all signals within the aa64–120 region were broadened beyond detection once a 1∶1 stoichiometry was reached, whereas for 14merS, equivalent signal loss occurred at 0.2∶1 RNA∶protein ratio. Notably, the ALYREF-binding region aa103–120 [30] was affected most severely by the addition of RNA, suggesting that the RNA and ALYREF-binding sites partially overlap. To determine if this ALYREF binding region is sufficient for specific RNA binding, the short ORF57^103–120^ peptide was titrated with 7merS, however no NMR signal broadening occurred and only small signal perturbations (under 0.04 ppm) were observed even with a 3-fold excess of RNA (Fig. S1C). Signal perturbation mapping therefore suggested a specific RNA binding site encompassing aa64–120 within ORF57^8–120^, whilst also showing the ALYREF-binding region aa103–120 located within this site is not sufficient for the recognition of specific viral RNA. Fluorescence measurements were also used to estimate the *K*_d_ for 14merS binding as 7.57±0.06 µM, compared to 38.8±0.6 µM for 7merN (Fig. S7A,B).

Unexpectedly, the addition of both specific and non-specific RNA oligos caused small NMR signal shifts in the acidic region of ORF57^8–120^ (aa10–40). We could not observe intermolecular NOEs between RNA and protein for the definitive binding epitope mapping, therefore, to separate possible indirect effects of conformational changes on signal shifts brought about by RNA binding and identify direct points of contact, we used RNA→ORF57 cross saturation transfer (ST) experiments [37], [39] (see Fig. S1D). These experiments report directly on the spatial proximity of RNA moieties to NH groups of individual amino acid residues (contact distance <5 Å), and provide essentially the same type of information as traditional RNA-protein cross-linking assays, but in a site-specific manner. A sample containing ^15^N-labelled ORF57^8–120^ : RNA 7merS in a ratio of 1∶0.5 was prepared (higher RNA concentrations prevented measurements due to excessive signal broadening). Selective saturation of RNA signals with a series of radiofrequency pulses resulted in a significant decrease in signal intensity of the backbone amides in ^1^H-^15^N correlation spectra (relative to the reference spectrum with off-resonance saturation) of mainly aa107–120 and aa81–92, and to a lesser extent, aa94–105, and even less, aa64–79 (Fig. 1A and Fig. S1E). (The typical effects of RNA→ORF57 saturation transfer on selected example signals from amides non-adjacent and adjacent to RNA are shown on the bottom right traces of the Figure “Typical effects of complex formation and RNA→protein ST” introduced later in the Results section.) The RNA→ORF57 ST experiment was repeated as a control with non-specific RNA 7merN, but no site-specific saturation transfer, and hence no direct interaction, was detected even when using a 2-fold excess of RNA (Fig. 1A and Fig. S1E). Based on the results of saturation transfer mapping, which are also in line with signal perturbation mapping, we conclude that ORF57^8–120^ contacts the specific RNA motifs directly using primarily its regions aa107–120 and aa81–92, with additional contribution from residues within aa94–105 and aa64–79. No significant binding was detected with non-specific RNA of similar length.

Previously we mapped aa103–120 as the ALYREF interaction site in HVS ORF57 [30]. Here, residues within the same region were also implicated in binding with a specific RNA motif. Given the multi-functional importance of this region, we endeavored to characterize it structurally. The secondary structure prediction algorithms Psipred [40] and Agadir [41] suggest ORF57 aa108–118 should be α-helical. Our experimental NMR data, namely dihedral angles derived from TALOS+ [42], ^15^N[^1^H] NOE experiments, and presence of characteristic *i* to *i+3* NOEs for a shorter peptide ORF57^103–120^ (see Fig. S2), also all demonstrate that the ORF57^8–120^ site aa107–118 exists in α-helical conformation; therefore this region was named “R-b helix”.

### Solution structure of the ALYREF-ORF57 interaction interface

**Figure 2:**
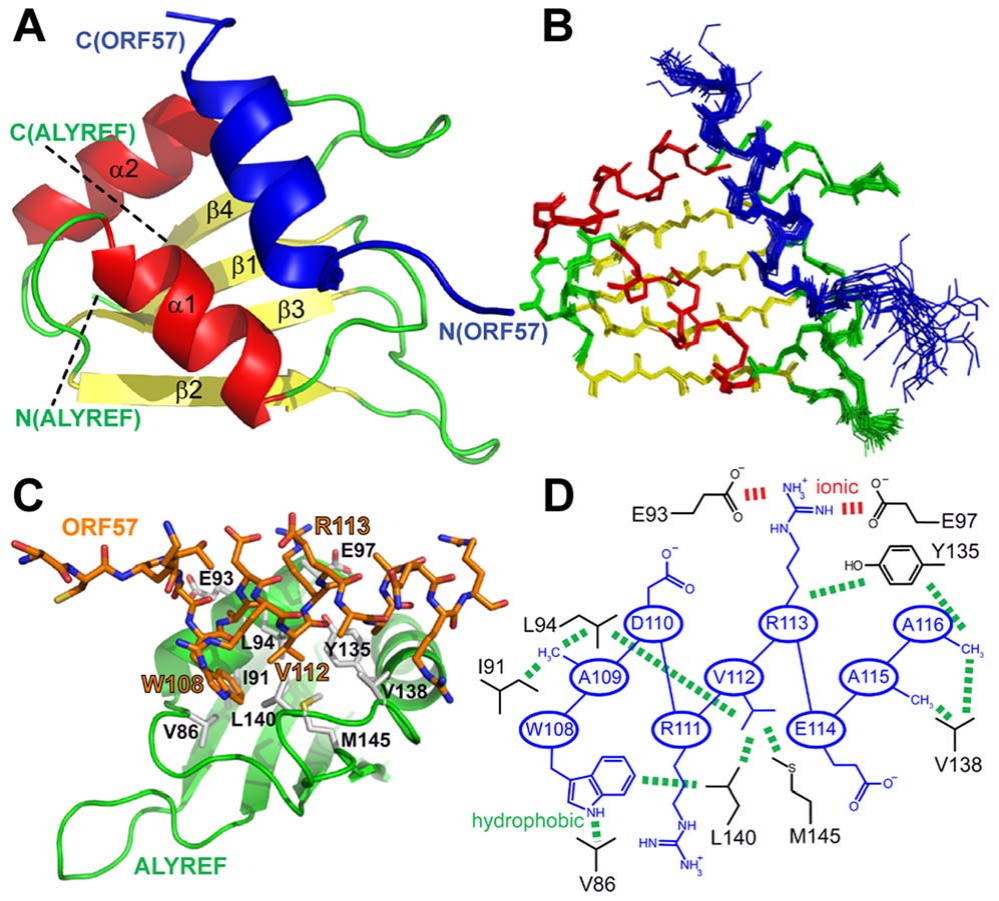
Structure of the ALYREF – ORF57 complex. (A) Ribbon representation showing ORF57 colored blue, and ALYREF RRM colored green, red and yellow for looped, α-helical and β-sheet regions, respectively. Positions of N- and C-termini of polypeptide chains are labeled. (B) Overlay of 20 lowest energy structures with backbone shown in the same orientation. The best-fit superposition is made using heavy backbone atoms of structurally defined regions aa74–152 of ALYREF and aa108–119 of ORF57. Color-coding is the same as on panel A. (C) Alternative view of ALYREF^54–155^-ORF57^103–120^ complex showing the hydrophobic sidechains involved in the interaction. (D) Schematic of the ALYREF and ORF57 binding site. ORF57 residues are colored blue and ALYREF in black; hydrophobic and electrostatic interactions are indicated by green and red dashes, respectively.

**Figure 3:**
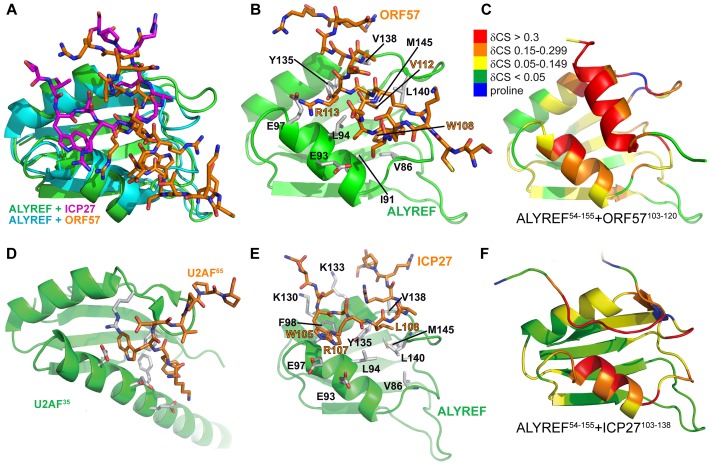
Comparison of ALYREF^54–155^-ORF57^103–120^ with ALYREF^54–155^-ICP27^103–138^ and U2AF complex structures. (A) Overlay of the RRM domains of ALYREF in complex with ICP27^103–138^ (green and magenta, PDB code 2kt5 [30]) and ORF57-bound ALYREF determined here (cyan and orange), demonstrating the shift in α-helix 1 position. (B) ALYREF^54–155^ in complex with ORF57^103–120^, determined here. (C) Backbone amide weighted chemical shift changes (δCS) in the ALYREF^54–155^-ORF57^103–120^ complex are emphasized by color. δCS>0.3 are red, 0.15–0.299 orange, 0.05–0.149 yellow, prolines are blue and regions unaffected are green. (D) U2AF^35^ in complex with U2AF^65^ (PDB code 1jmt, [50]). (E) ALYREF^54–155^ in complex with ICP27^103–138^, previously determined (pdb 2kt5). (F) Backbone amide weighted chemical shift changes (δCS) in the ALYREF^54–155^-ICP27^103–120^ complex with the same coloring as panel C.

Previously the structure of the complex of ALYREF fragment aa54–155 (ALYREF^54–155^) with ORF57^8–120^ could not be determined due to an unfavorable chemical exchange regime, causing signal broadening for the interacting residues [30]. In view of the importance of the aa103–120 region for both ALYREF and RNA binding, and differences in local structure of ALYREF-binding regions of ICP27 [30] and ORF57, we pursued the structure of the ORF57-ALYREF complex interface. We employed a short ORF57^103–120^ construct, which displayed much improved spectra and less exchange behavior (Fig. S3). The atomic resolution structure of the ALYREF^54–155^ - ORF57^103–120^ complex was determined using a total of 2427 non-redundant NOEs, 122 of which were intermolecular (Table 1 and Fig. S4). Previous signal perturbation mapping indicated that ORF57 aa103–120 comprise the ALYREF-binding site [30]; the new data defined the site more precisely as aa106–120 (Fig. S3C). Within the complex, the ORF57 peptide is α-helical for aa108–119, contacting the loops L1 and L5 on the α-helical face of ALYREF (Fig. 2). The binding site on ALYREF is composed of a hydrophobic patch formed by the sidechains of L82, V86, L94, Y135, V138, L140 and M145, with E93 and E97 contributing to ionic interactions (Fig. 2D). The aromatic sidechain of W108^ORF57^ is positioned at one end of this hydrophobic patch of ALYREF in close proximity to the sidechain of V86 (Fig. 2D). The majority of the remaining hydrophobic contacts of ORF57 are formed by V112 and the aliphatic part of the R113 sidechain, with A109, A115 and A116 also contributing. The positive charged R113^ORF57^ sidechain is positioned between ALYREF residues E93 and E97 which are therefore likely to form salt bridges. The structure reveals unexpected differences in binding conformations and molecular recognition of two functionally-similar viral adaptor proteins, HVS ORF57 and HSV-1 ICP27, on essentially the same site on ALYREF (Fig. 3 and Fig. S3D), despite the presence of deceptively similar recognition triads identified earlier [30].

**Table 1:** NMR calculation statistics for an ensemble of the 20 lowest energy structures of ALYREF fragment (ALYREF^54–155^) bound to ORF57^103–120^ (PDB code 2YKA).

Total number of NMR restraints	2629
Number of NOE restraints	2427
Intra-residue	534
Sequential	651
Medium range (2≤i≤4)	448
Long range intramolecular (5≤i)	794
Intermolecular	122
Dihedral	164
Hydrogen bonds	38
Mean number of NOE violations >0.1 Å	0.0108±0.0010
Mean number of dihedral violations >5°	0.3144±0.0375
Mean Cyana target function, Å^2^	1.58±0.18
Coordinate precision, Å	
RMSD (ALYREF^74–152^+ORF57^106–120^)	
Backbone	0.26±0.07
Heavy atom	0.81±0.08
RMSD (ALYREF^74–152^)	
Backbone	0.21±0.05
Heavy atom	0.73±0.06
RMSD (ORF57^106–120^)	
Backbone	0.29±0.13
Heavy atom	1.01±0.19
Ramachandran plot (ALYREF^74–152^+ORF57^106–120^), % residues in regions:	
Favored	79.8
Additional	20.2
Generous	0.0
Disallowed	0.0

### Viral RNA binds to ALYREF only weakly, as detected by NMR

ALYREF is known to bind RNA weakly and non-specifically, primarily using its flexible N- and C-terminal domains [7]. Using NMR signal perturbations, non-specific 15-mer RNA (15merN) oligonucleotide binding to the ALYREF fragment aa1–155 (ALYREF^1–155^) was previously mapped to RGG motifs situated within its unstructured N-terminus and also to loops L1 and L5 of the RRM domain [10]. To address how well ALYREF binds to the viral mRNA specifically recognized by ORF57, we firstly explored how ALYREF binding to RNA depends on the length of the viral oligo sequence. Chemical shift mapping was carried out with [^15^N]-REF^1–155^ using equimolar 7merS or 14merS (Fig. 1B). The data indicated that the short RNA 7merS causes small perturbations almost exclusively within the RRM domain in loops L1, L3 and L5, whereas the longer 14merS caused signal broadening within the N-terminal aa12–48 along with minor shift changes within the RRM. The extent and location of signal changes is similar to that observed previously using non-specific 15-mer RNA (15merN) [10], suggesting that ALYREF itself cannot discriminate between viral and non-viral RNA, and binds it only weakly. Saturation transfer experiments confirmed that 14merS RNA contacts ALYREF in the N-terminal region containing RGG motifs (Fig. 1B and Fig. S1F), at the same site where non-specific RNA binding occurs. The measurement of the *K*_d_ for ALYREF^1–155^ interaction with RNA oligonucleotides using fluorescence unfortunately could not be completed due to increase in sample turbidity upon RNA addition, likely caused by non-specific protein aggregation. From NMR titration data the lower-limit *K*_d_ estimates for 7merS and 14merS binding were >100 µM and >50 µM, respectively. These values are significantly higher than the values characterizing the specific binding of the 14merS to viral ORF57^8–120^ (7.57 µM, Fig. S7A), and closer to the *K*_d_ for non-specific binding of 7merN to ORF57^8–120^ (38.8 µM, Fig. S7B). Overall, these estimates show that the viral RNA motif is specifically recognized and binds with viral ORF57^8–120^ but not ALYREF^1–155^, suggesting that in the cell the viral mRNA would be initially preferentially recognized and bound by viral ORF57.

### Competition between ALYREF and RNA for binding to R-b helix of ORF57 monitored by IDIS NMR experiments

As shown above, the ORF57 region aa106–120 is involved in specific viral RNA binding, but it can also be utilized for ALYREF binding. This raises the question: which binding partner does this particular region select when all three components are present? Here we used solution NMR experiments to investigate *directly* if these local interactions are indeed competitive, and which of these is stronger and hence is preferentially selected when the ternary complex is formed. Residue-specific signal changes in the ORF57-ALYREF complex upon addition of unlabelled RNA were monitored using an IDIS-TROSY experiment [38], which allows the observation of separate ^1^H-^15^N-correlation spectra of two differentially-labeled proteins in the same sample. At a stoichiometric 1∶1 ratio of [^15^N,^13^C]- ORF57^8–120^ and [^15^N]-REF^1–155^, the backbone amides at the protein-protein interface are exchange broadened (including aa106–120 of ORF57), however signals from other regions of both proteins are clearly observable, and their pattern is characteristic of ORF57-ALYREF complex formation. Having been assigned to particular amino acid residues, they were able to report site-specific changes in the protein-protein interactions in response to ligand binding (Fig. 1, Fig. S5 and Fig. S6).

In an initial experiment, a stoichiometric equivalent of a shorter specific RNA 7merS was added to the differentially-labeled ORF57-ALYREF complex. In contrast to the substantial broadening of aa64–120 observed on addition of 7merS to free ORF57^8–120^, no broadening and only small shifts in ORF57 signals were observed when ALYREF was present (Fig. 1). This indicates that ALYREF reduces ORF57 binding to specific viral RNA, protecting its binding site, aa106–120. A control experiment using ALYREF^54–155^, which lacks the N-terminal RNA binding site, produced similar results, also suggesting that the region aa106–120 of ORF57 has higher affinity for ALYREF than for a specific RNA oligo 7merS (Fig. S6). The ALYREF signal changes induced by the addition of 7merS were marginal. In a related experiment, to directly follow the displacement of RNA from ORF57, one equivalent of the 7merS was added to [^15^N]-ORF57^8–120^ causing substantial signal broadening in aa64–120. Then one equivalent of [^15^N,^13^C]-REF^1–155^ was added to the same sample, resulting in recovery of all ORF57 signals except for those which became instead involved in the ALYREF interaction (aa106–120) and remained broad. IDIS-TROSY spectra of both ALYREF and ORF57 confirmed formation of the protein-protein complex as the fingerprint pattern of observable ORF57 signals was consistent with ORF57 bound to ALYREF, but not RNA. These direct experiments demonstrate that virus-specific RNA is displaced from ORF57 aa106–120 by the competitive binding of ALYREF to this region, and not by the preferential binding of RNA to ALYREF.

### RNA binding within the ternary RNA-ALYREF-ORF57 complex mapped by ST IDIS NMR experiments

**Figure 4:**
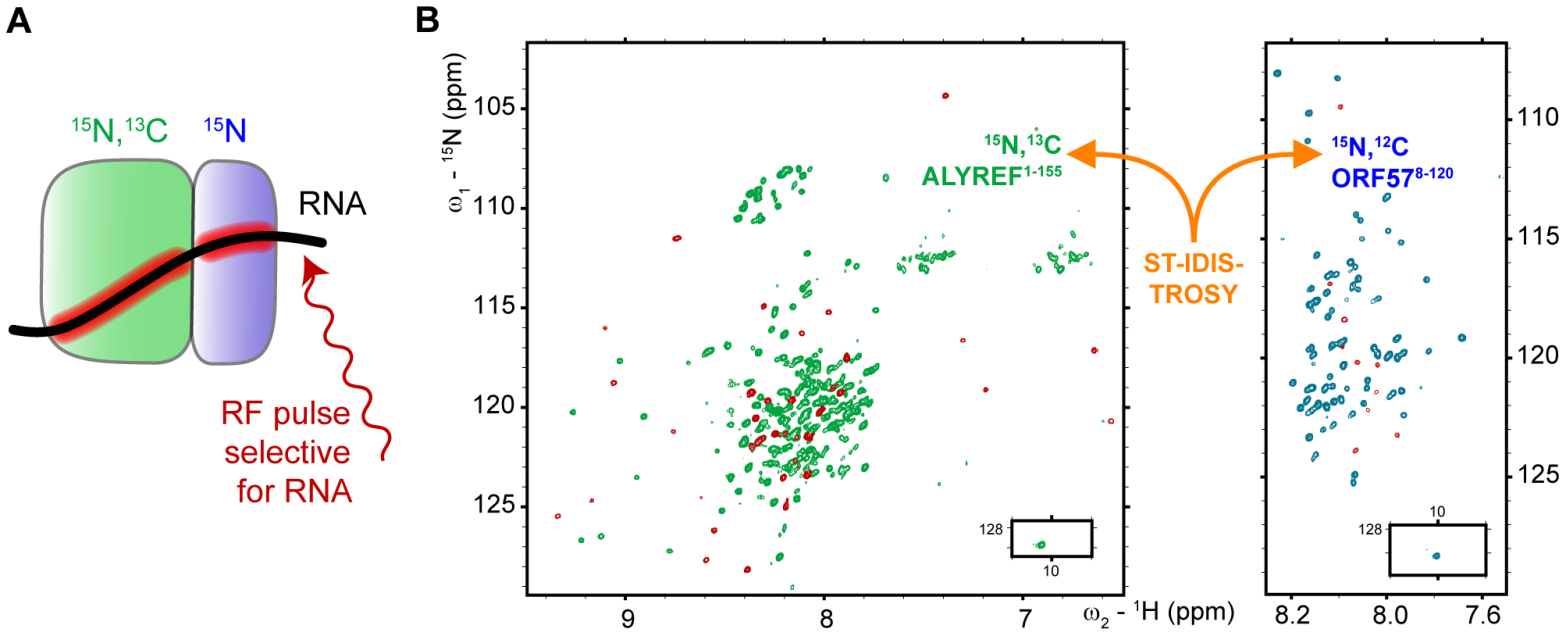
Obtaining site-specific information on RNA binding to protein-protein complex. (A) Scheme illustrating the principle of the ST-IDIS-TROSY method proposed here. RNA is added to a differentially labeled pair of interacting proteins, and selective saturation of RNA protons by RF pulses is transferred through space to the adjacent amides within both proteins in the complex. (B) The example of the resultant ST-IDIS-TROSY spectra: signals from amide groups situated within 5 Å of the RNA are selectively weakened (colored red), as detected independently and simultaneously in the spectra of both proteins.

**Figure 5:**
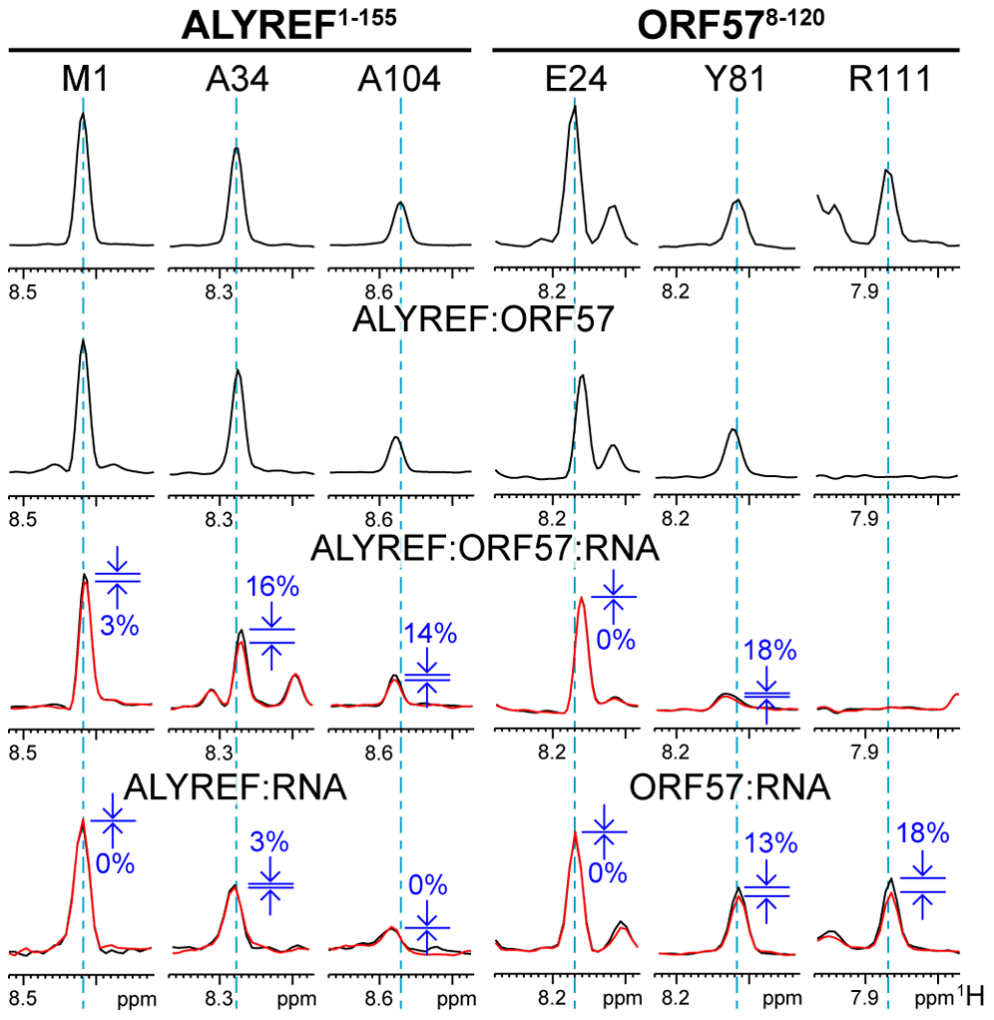
Typical effects of complex formation and RNA→protein ST on selected signals of ALYREF^1–155^ and ORF57^8–120^. The ^1^H dimension slices through ^1^H-^15^N-correlation spectra are displayed for three representative signals of each protein, on the left for ALYREF and on the right for ORF57. The residue assignments in the free form are labeled at the top, and same signals are shown below each other for different complexes, as indicated. First type of signal (ALYREF^M1^ and ORF57^E24^) is not significantly affected by any complex formation, or ST. Second type (ALYREF^A34^ and ORF57^Y81^) is not affected much by protein and marginally affected by RNA binding, but is altered or displays significant ST effect in the ternary complex (percentage drop in signal intensity is indicated in blue, and ST spectral traces shown in red). These are residues likely contributing to cooperative ternary complex formation, forming contacts with RNA. Third type of signals originates from the structured regions of proteins (ALYREF^A104^ and ORF57^R111^). ALYREF^A104^ signal is not significantly affected in protein-protein complex, but shows significant increase in ST effect in the ternary complex, suggesting that ORF57 recruits RNA to the proximity of this residue. ORF57^R111^ signal is broadened beyond detection in protein-protein complex, and remains broadened in the ternary complex. For this signal strong ST effect is observed when in complex with RNA. This residue is involved in initial viral RNA recognition, but then RNA is displaced from this site by ALYREF binding.

As the short RNA 7merS cannot be bound efficiently by the ORF57-ALYREF complex, subsequent experiments were performed using a longer specific RNA 14merS. Importantly, as evidenced by the presence of a large number of relatively sharp signals from amide groups of both proteins (Fig. 4, Fig. 5, Fig. S5, Fig. S6), the complexes formed retained a high degree of flexibility, even for residues directly involved in interactions. There were no NOE signals observed between RNA and proteins, making it impossible to apply standard techniques for full 3D structure determination of the ternary complex. Therefore, to obtain information regarding the spatial organization of this largely fluid assembly, a saturation-transfer version of isotopically-discriminated TROSY [38] experiment (ST-IDIS-TROSY) was created and used to detect directly, in residue-specific manner, where exactly RNA contacts the ALYREF^1–155^ and ORF57^8–120^ in the ternary complex (Fig. 4). A sample was prepared containing a 1∶1∶1 mixture of [^15^N,^13^C,^2^H]-ORF57^8–120^, [^15^N,^2^H]-REF^1–155^ and non-labeled 14merS (∼40 kDa complex in total). Protein deuteration was used to improve the quality of spectra and reduce possible artifacts due to spin diffusion effects [37]. RNA proton signals were selectively saturated by radiofrequency pulses [37], [39], and changes in IDIS-TROSY [38] peak intensities were monitored to reveal which amide groups are situated in close proximity (<5 Å) to RNA moieties, observing fingerprint spectra from both proteins at once (Fig. 4B). The examples of typical changes in individual signals (from interacting and non-interacting sites) in response to complex formation and saturation transfer are shown for illustration on Fig. 5, with residue-specific results presented in Fig. S1G,H, and an overview is included in Fig. 1. When the saturation transfer effect was initially calculated from the ratio I^5.85^/I^21.0^ obtained with on-resonance ribose proton (5.85 ppm) and off-resonance (21.0 ppm) saturation, we noticed a significant amount of non-specific saturation transfer to virtually all serine residues in ORF57. We explained that by the inadvertent saturation of serine hydroxyl groups. To compensate for this effect, we have used two different saturation schemes. In the first scheme, we selectively saturated two RNA resonances (moieties) with similar chemical shifts (5.75 and 5.85 ppm), and calculated the ratio of signal intensities I^5.75^/I^5.85^ (Fig. S1G). If one of the saturated RNA moieties is positioned closer to a protein amide group (and within 5 Å) than the other, then the amount of cross-saturation transfer from them to this amide will not be equal. Hence, where the ratio I^5.75^/I^5.85^ deviates from unity, it highlights residues adjacent to RNA. The close positioning of saturating frequencies on the other hand should cross-saturate broad hydroxyl signals to a similar extent, compensating for this artifact. In the second scheme, the on-resonance saturation was centered at 12.0 ppm and off-resonance at 21.0 ppm, and I^12.0^/I^21.0^ ratio calculated (Fig. S1H). The RNA signals at 12.0 ppm were broad and not observable, but this frequency was chosen as it is characteristic for RNA imino protons. Both saturation schemes led to similar mapping results: whereas many protein amide resonances remained unaffected by the RNA signal saturation, several regions in ALYREF and ORF57 in the ternary complex were clearly highlighted (Fig. 1 and Fig. S1G,H). The most pronounced ST effect was observed for the arginine-rich N-terminal region of ALYREF aa24–48, with parts of the RRM domain also affected (Fig. 1B). The region aa79–100 within ORF57 was also highlighted by saturation transfer, as seen by the deviations of the I^5.75^/I^5.85^ and I^12.0^/I^21.0^ ratios from unity. The increase in estimated error margins within the regions affected by ST (Fig. S1G,H) is explained by signal broadening, leading to a reduction of signal intensities for amides in contact with RNA. The presence of ALYREF in the sample clearly reduces the size of the ORF57 site available for RNA binding (Fig. 1A). In the presence of ORF57, the saturation transfer from RNA 14merS to ALYREF becomes more pronounced (i.e., larger deviation of I^freq1^/I^freq2^ from unity), suggesting that RNA is retained by ALYREF within the ternary complex more efficiently than by ALYREF alone.

The NMR experiments therefore all indicate that ALYREF partially displaces the viral RNA initially bound specifically to ORF57, but retains it within the complex. In the ternary complex ORF57 aa106–120 directly interacts with the ALYREF RRM, whereas flexible flanking regions of ALYREF (aa24–48) and ORF57 (aa81–92), and to lesser extent, parts of helix 2 of the ALYREF RRM, jointly keep hold of the viral RNA molecule. Interestingly, amide signals from flexible protein regions which become involved in direct contacts with RNA (as evidenced by RNA-protein saturation transfer), are only partially broadened in the complex. They had intensities higher than signals from the folded regions, but lower than signals from the unfolded non-interacting regions (examples of this behavior can be seen in Fig. 5 and Fig. S5). This suggests that the interaction with RNA in these conditions was somewhat transient and did not lead to the formation of a rigid 3D structure.

### Cooperativity of RNA - ALYREF - ORF57 complex assembly studied by fluorescence

**Figure 6:**
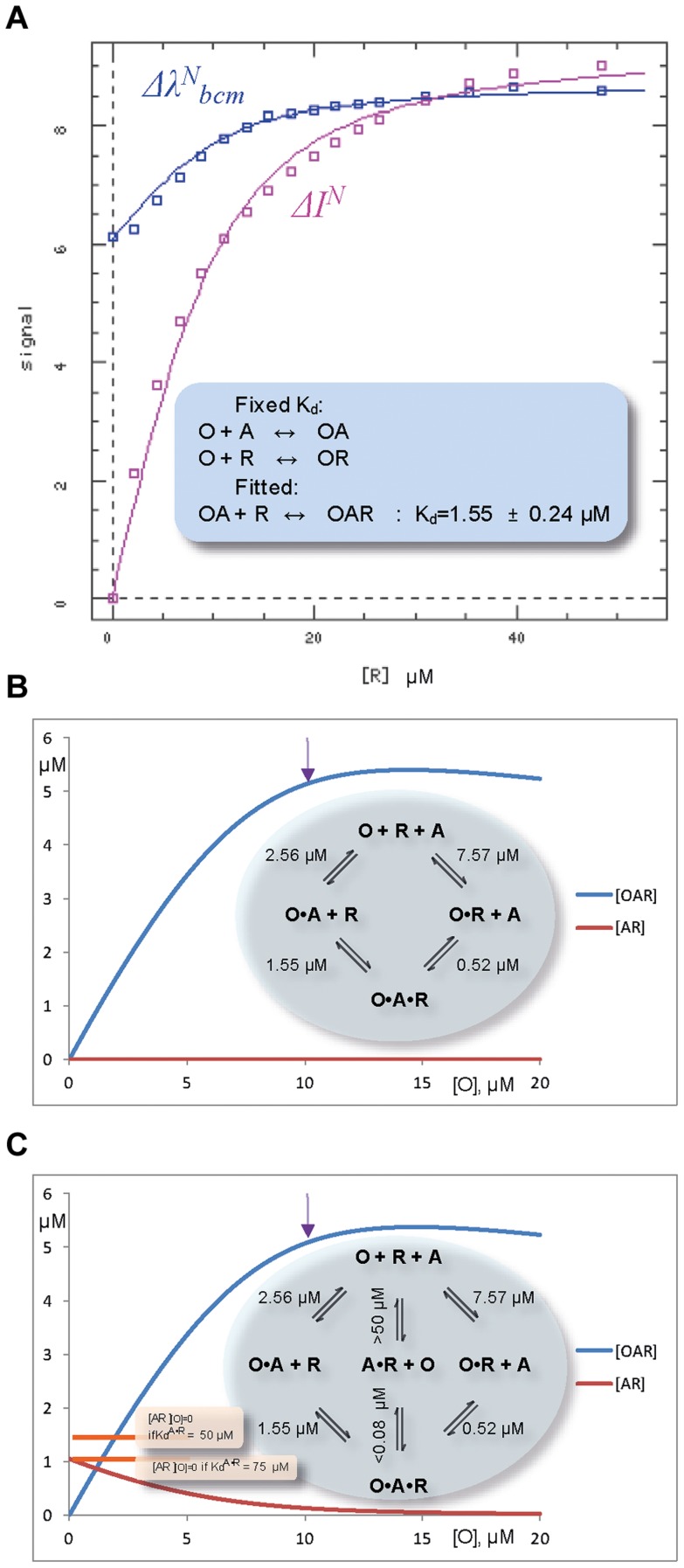
Fluorescent studies and simulations of the ternary complex formation between ORF57, O, fragment of viral RNA, R, and ALYREF, A. (*A*) Simultaneous non-linear fit of normalized fluorescence-derived parameters *Δλ^N^_bcm_* (blue shift of emission signal) and *ΔI^N^* (fluorescence quenching) to the non-redundant three-equation model using DynaFit software, to obtain *K_d_* for the ternary complex. The experimental values of thus determined *K_d_^OA+R^*, as well as *K_d_*'s for other complexes measured earlier, are summarized on two illustrative thermodynamic cycles for the ternary complex assembly presented on panels *(B)* and *(C)*. Simulations (using COPASI software [67]) for these two possible cycles illustrate an increase in the concentration of ternary ORF57-RNA-ALYREF complex when ORF57 is added to 10 µM equimolar mixture of ALYREF and RNA, assuming the simplest four-state equilibrium model (*B*), or six-state model which additionally takes into account weak nonspecific ALYREF-RNA binding (*C*). The arrow marks a point where all the components of ternary complex are present in equimolar amounts. The presence of equimolar ORF57 significantly increases the concentration of RNA in complex with ALYREF (i.e., [OAR] vs [AR]_[O] = 0_). The baseline concentrations [AR]_[O] = 0_ are indicated on the panel (*C*) on the left, assuming two different conservative estimates for values of *K*_d_ for nonspecific ALYREF-RNA binding.

Fluorescence measurements were used to quantify the overall strength of the ALYREF^1–155^ and ORF57^8–120^ interaction in the absence and presence of a specific fragment of viral RNA. Both protein constructs possess tryptophan residues, one of these (W108 of ORF57) forms part of their binding interface, and is buried upon protein complex formation. Control experiments showed that ALYREF^1–155^ and ORF57^8–120^ both have a fluorescence intensity maximum at 355 nm which is not shifted by 14merS RNA addition (however, ALYREF samples become turbid due to non-specific aggregation, complicating measurements). The formation of equimolar ALYREF^1–155^ - ORF57^8–120^ complex leads to a blue shift of the emission maxima of the sample (Fig. S7C), in agreement with burial of the tryptophan sidechain in a hydrophobic environment. The blue shift, quantified by λ_bcm_, becomes more pronounced with increasing concentrations of equimolar ALYREF^1–155^ - ORF57^8–120^ in the sample, allowing an estimation of the apparent *K*_d_ for this interaction as 2.56±0.20 µM (Fig. S7C). Addition of 14merS to pre-mixed 10 µM equimolar ALYREF^1–155^ - ORF57^8–120^ complex caused both a decrease in fluorescence intensity (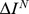, reflecting the change from binary protein-RNA to ternary complex formation), and a blue signal shift (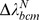, reflecting the change from binary protein-protein to ternary complex formation). Non-linear fit of the two dependencies together to the three-equation equilibrium model using DynaFit software [43] (see Fig. 6A) allowed an estimation of the apparent macroscopic *K*_d_ for the RNA binding to ORF57-ALYREF as 1.55±0.24 µM. The overview of *K*_d_'s determined for this simplest equilibrium model for ternary complex formation [44] is presented on a thermodynamic cycle in the inset of Fig. 6B. The estimated value of *K_d_^OR+A^* = 0.52 µM for binding of ALYREF to ORF57-RNA complex can be inferred from the thermodynamic equilibrium considerations [44]. As the measured *K_d_* values for the formation of binary complexes are significantly higher than for the ternary complexes (e.g., *K_d_^O+R^* = 7.57 µM>*K_d_^OA+R^* = 1.55 µM), the assembly shows clear-cut cooperative behavior [44]. These results therefore reveal that the ternary complex formation, leading to introducing RNA to ALYREF, is thermodynamically driven by the overall cooperativity. Further analysis of this simplest equilibrium model using COPASI simulations illustrates a dramatic increase in the population of ALYREF molecules bound to RNA when the ORF57 is present (Fig. 6B). We have also run COPASI simulations for an extended binding model, where a very weak non-specific binding of RNA to ALYREF (with estimated *K_d_*>50 µM, see above) is taken into account (Fig. 6C), and two different *K_d_* values for non-specific binding are assumed for calculations as examples. The COPASI simulations for each model demonstrate that the presence of ORF57 in stoichiometric amounts significantly increases the concentration of ALYREF in complex with RNA (i.e., [OAR]), compared to the background level of non-specific ALYREF-RNA complex (i.e., [AR], Fig. 6B,C). Even with the most conservative estimates (assuming the lowest value of *K_d_* = 50 µM for non-specific ALYREF-RNA binding), for the concentrations used in this example the amount of virus-specific RNA in complex with ALYREF increases more than 3.6 times.

Interestingly, adding a large excess of RNA to the 2.5 µM ALYREF-ORF57 mixture displayed a more complex behavior of signal shift (Fig. S7D): the blue shift observed with only a small excess of RNA was partially reversed, consistent with protein-protein complex dissociating at higher RNA excess. Intuitively this result is expected if RNA over-saturates the binding site on ORF57, competitively displacing ALYREF from R-b helix. This competitive behavior at very high [RNA] cannot be adequately described by currently parameterized simple equilibrium models, such as shown on Fig. 6B,C, which only account for cooperativity.

The experimental fluorescence equilibrium binding data thus reveal the overall cooperativity in the ternary complex formation when the components are present at or near stoichiometric amounts, and support a role of ORF57 as an adaptor introducing RNA to ALYREF. The fluorescence data are also consistent with a local competitiveness of ORF57-ALYREF and ORF57-RNA interactions: this competitiveness becomes apparent at macroscopic (i.e., molecular) level only if RNA is in significant excess. These observations fit well with the NMR experiments which show the ability of ALYREF to partially displace RNA from R-b helix of ORF57, while forming ternary complex.

### RNA binding of ALYREF and ORF57 studied by UV cross linking

**Figure 7:**
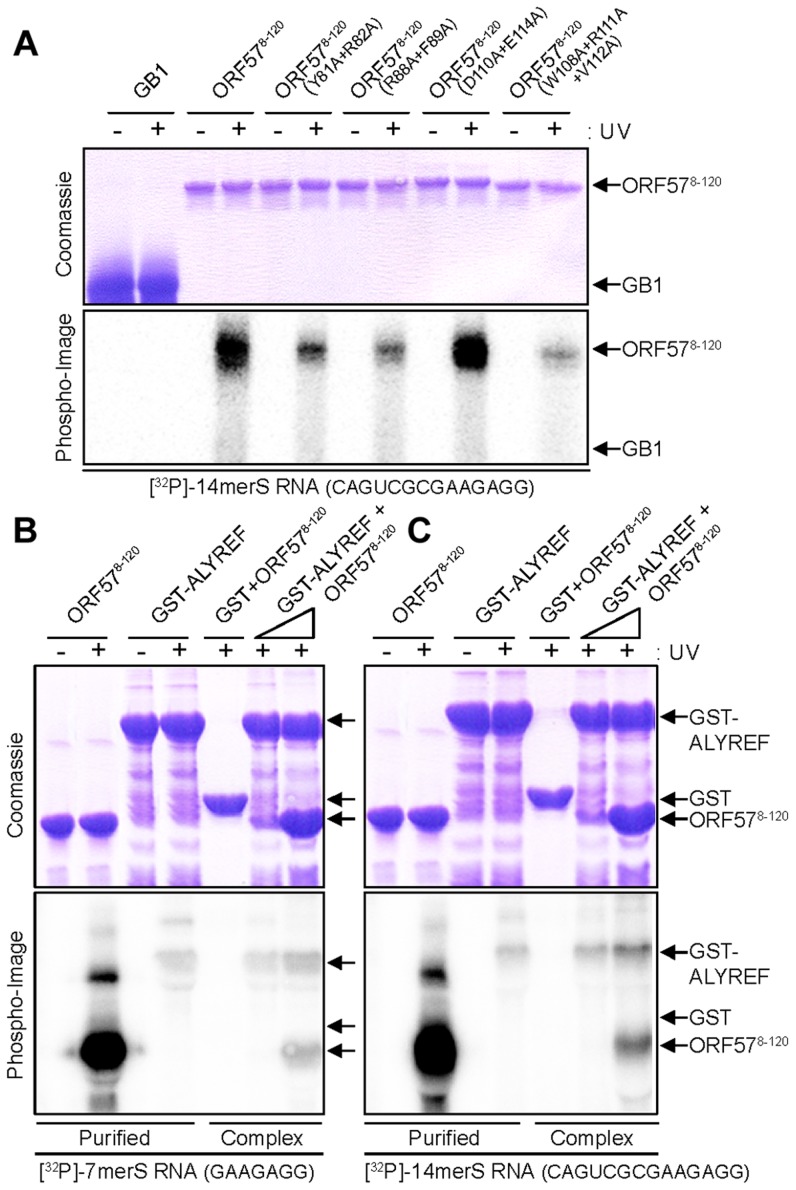
Probing ORF57-RNA binding by mutations and UV cross-linking, and ALYREF-ORF57-RNA remodeling assay. (*A*) Purified hexa-histidine tagged GB1 (negative control) or ORF57^8–120^ WT and point mutants (as labeled) were incubated with end-labeled 14merS RNA oligonucleotide before the mixture was subjected to UV cross-link (+) or not (−). Similarly in the remodeling assay, WT ORF57^8–120^ was incubated with end-labeled 7merS (*B*) or 14merS (*C*), before the mixture was added to purified GST-ALYREF immobilized onto glutathione coated beads. Purified complexes were eluted in native conditions and UV cross-linked (+) or not (−). All samples were finally analyzed on 15% SDS-PAGE stained with Coomassie blue and by PhosphoImaging.

The results of NMR mapping of RNA binding regions of ORF57^8–120^ were confirmed by UV cross-linking using purified protein and radio-labeled RNA oligonucleotide, performed as previously described [10]. The ORF57 mutants Y81A+R82A, R88A+F89A and W108A+R111A+V112A all significantly reduced the efficiency of cross-linking with RNA 14merS (Fig. 7A). Substitution of residues W108,R111,V112, which are the most important for ALYREF binding [30], also has the strongest reductive effect on RNA binding, confirming independently that the RNA- and ALYREF-binding sites overlap. The control mutation D110A+E114A marginally increases the efficiency of RNA cross-linking, this is likely to be due to reduction in electrostatic repulsion between this protein mutant and RNA.

To independently confirm the NMR observations in regard to RNA oligonucleotide binding with ORF57^8–120^, ALYREF^1–155^ and their complex, we performed *in vitro* reconstitution assays followed by UV cross-linking experiments. ORF57^8–120^ showed a strong RNA-binding activity for 7merS and 14merS in sharp contrast to GST-ALYREF which bound weakly with both RNAs (Fig. 7B,C). When ORF57^8–120^ was incubated with RNA prior to mixing with GST-ALYREF, followed by GST affinity purification of the resulting complexes and UV cross-linking, there was a drastic reduction of the RNA cross-linked to ORF57^8–120^ and a concomitant increase in the RNA cross-linked onto GST-ALYREF. Therefore the RNA-binding activity of ORF57^8–120^ is severely reduced upon interaction with ALYREF, whereas the amount of RNA in contact with ALYREF increases in the ternary complex. This independent data obtained at a molecular (i.e, macroscopic) level concurs fully with the NMR data obtained at a residue-specific level of detail. Interestingly, the increase in ALYREF-RNA cross-linking efficiency observed experimentally in the presence of ORF57 fits well with the numerical estimates using COPASI simulations shown on Fig. 6C.

### A molecular model for how viral mRNA is transferred from viral ORF57 to cellular ALYREF

**Figure 8:**
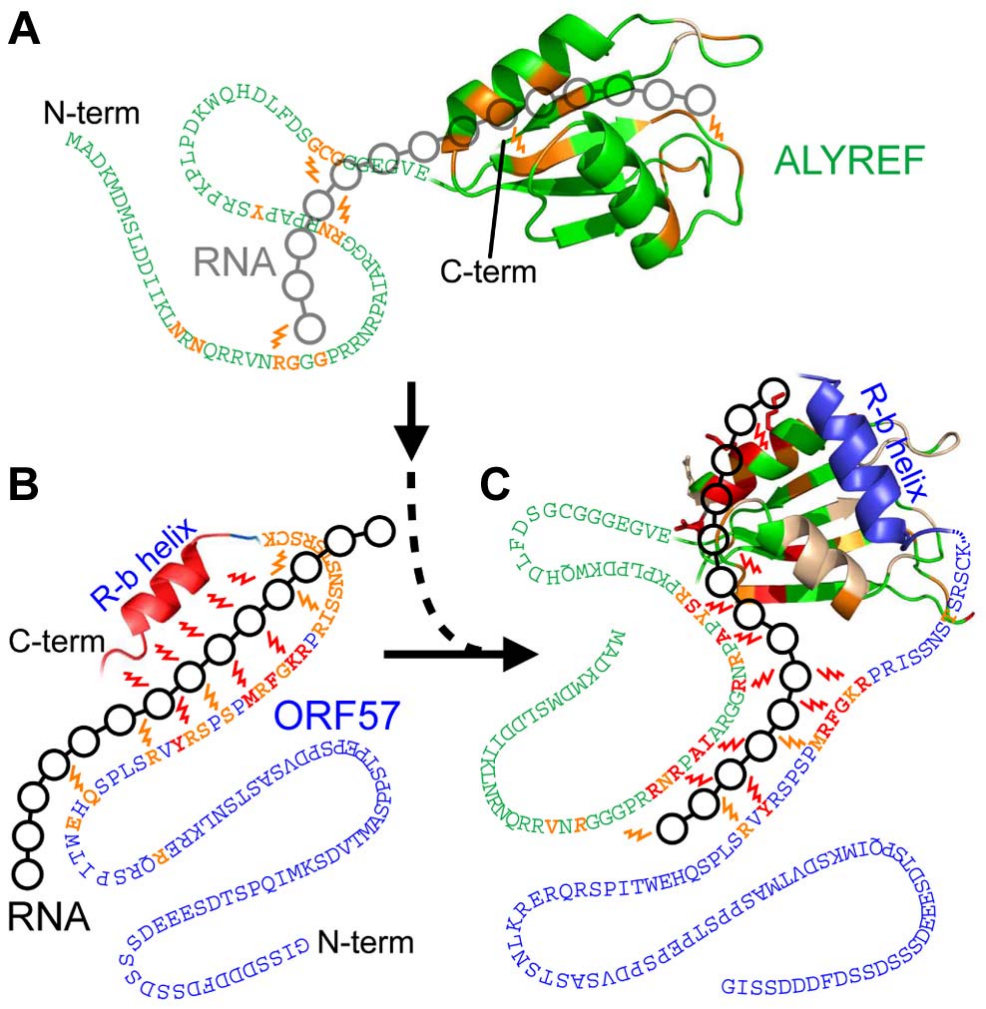
Model of the passage of RNA between ORF57 and ALYREF. Local protein interactions with RNA are detected by moderate (orange) and large (red) saturation transfer effects (represented by ‘lightning bolts’) observed by ST-HSQC or ST-IDIS-TROSY and mapped onto respective regions. Broadened residues are colored light-yellow. Linked black circles represent a position for transiently bound RNA. ALYREF (green) binds RNA 14merS weakly via its RRM and N-terminal regions (A), whereas ORF57 (blue) binds 14merS tightly mainly via the R-b helix and also the aa81–92 region (B). Interaction of ORF57-RNA complex with ALYREF partially displaces the RNA from the R-b helix, while RNA maintains contact with ORF57 aa81–92 and also forms new contacts with ALYREF's aa22–48 and helix-2 of the RRM domain (C). The RNA contacts with ALYREF within the ALYREF-ORF57-RNA ternary complex are more abundant than for just ALYREF-RNA, and thus ORF57 enhances the interaction of viral RNA with ALYREF.

The combination of structural and interaction data allows us to suggest a model that explains how the adaptor protein ORF57 from HVS introduces viral mRNA to cellular mRNA export factor ALYREF, functioning as a molecular “hijacker”. As previously noted, ALYREF itself binds mRNA weakly and non-specifically, and cannot discriminate between cellular and viral transcripts, needing other proteins to recruit mRNA and strengthen this binding to a functionally significant level. This non-specific binding can be observed and mapped here by weak saturation transfer from RNA to protein (Fig. 1). In its free form the N-terminal region aa8–120 of ORF57 is flexible and mainly unstructured, apart from the short α-helix aa108–118 which we named R-b helix. It is anticipated that the positively charged region aa61–120 interacts transiently with the negatively charged part of the ORF57 polypeptide chain aa12–28, keeping the molecule in a loosely “closed” conformation. When the specific viral mRNA motif binds, it is recognized by the extensive ORF57 region aa64–120 which comprises R-b helix (see Fig. 8). On its own, R-b helix is unable to bind RNA, but its presence, as well as the presence of the flanking region, are essential. It can be envisaged that the RNA-binding region of ORF57 may form a hairpin or other compact structure to offer an extensive network of contacts recognizing and holding the viral RNA molecule (Fig. 8).The fragment aa64–120, which is rich with arginines, serines and aromatic residues, forms direct contacts with RNA, but without forming a stable 3D structure, and this RNA binding is expected to release the negatively-charged N-terminal part of ORF57. ORF57 is not able to recognize and bind mRNA which lacks specific viral motifs, ensuring that this viral adaptor selects only viral transcripts for further export. Although the R-b helix of ORF57 participates in recognition and binding of viral mRNA, it has much higher affinity for ALYREF binding. Therefore in the presence of ALYREF (see Fig. 8), the R-b helix is released from RNA and binds the RRM domain of ALYREF instead. However at that point the adjacent flexible regions of both ORF57 and ALYREF, which are also involved in RNA binding, are brought together, and the viral RNA molecule is not released but is held in place by the synergetic action of these flanking regions (Fig. 8). The overall cooperativity of ternary complex assembly is demonstrated by the fluorescence measurements and supported by the remodeling assay, while the local competitiveness of RNA and ALYREF binding to R-b helix is directly demonstrated by the NMR data, and additionally supported by the fluorescent measurements showing ternary complex dissociating when over-titrated with RNA. The RNA binding within the stoichiometric ternary complex is mapped using direct NMR measurement of spatial proximity of individual amino acid residues of both ORF57 and ALYREF to RNA (Fig. 8). When the ternary RNA-ORF57-ALYREF complex is formed, the N-terminal regions of both ORF57 and ALYREF in contact with RNA retain significant flexibility (as evidenced by NMR signal shapes and lack of RNA-protein NOEs). The main NXF1-binding region of ALYREF aa15–36 [5], [10] partially overlaps with region involved in viral mRNA binding, and remains sufficiently exposed. This allows us to speculate that at the next stage of the pathway the NXF1 would bind to this region, partially displacing the viral mRNA, which however will be held in the vicinity by the rest of the binding site, formed by synergetic interactions of ALYREF and ORF57. ALYREF binding would then help switch NXF1 into the high-affinity RNA binding mode, forcing it to accept viral mRNA for export via the nucleopore, using the host pathway. We suggest that the presence of partially-overlapping binding sites, and a combination of competitive interactions at the level of specific sites, and cooperative binding at the macroscopic level, may provide a general molecular mechanism for targeted successive RNA transfer between protein molecules along the export pathway.

## Discussion

The functional role of the viral protein ORF57, to introduce specific viral RNA to the cellular protein ALYREF, thereby facilitating the export from the nucleus of unspliced viral mRNA via the NXF1-dependent cellular pathway, is fulfilled via several critical properties. First, ORF57 must recognize a specific viral mRNA, which contains characteristic sequence motifs, and ignore cellular mRNA. Second, it should interact with cellular ALYREF, and induce binding of a specific viral mRNA to ALYREF, allowing ALYREF then to pass it over to NXF1 at the next stage of the pathway. Therefore, the ability to recognize and reversibly bind RNA is crucial for correct and efficient functioning of viral ORF57 as a “molecular highjacker” of the host cell pathway. Here, we have reconstituted in vitro and studied in detail the function of the core responsible for the recruitment of viral RNA to ALYREF, using the essential protein fragments of ORF57(aa8–120) and ALYREF(aa1–155) which interact with each other and with viral RNA. A range of experimental approaches was used to reveal the molecular mechanism of viral RNA recognition and following transfer from ORF57 to ALYREF with atomic-to-residue-level resolution.

### Functional importance of RNA recognition region of ORF57

The position of viral mRNA binding sites on ORF57 was previously broadly localized to aa8–120 [20], [21]. This region is largely unstructured, and contains multiple arginines between residues 62 and 120 which may potentially mediate RNA binding, although this was not confirmed previously. Here, we used NMR to characterize the binding sites more precisely. To directly identify the residues in proximity to RNA we employed RNA→protein cross-saturation transfer experiments [37], [39] which revealed two main ORF57^8–120^ regions in direct contact with RNA, aa107–120 and aa81–92, with residues from aa94–105 and aa64–79 also contributing. Although the general “polyelectrostatic effect” [45] is expected to attract mostly negatively-charged RNA to positively-charged regions and thus explain the weak RNA-binding affinity of ALYREF, the amino acid sequence determinants of a sequence-specific RNA recognition by viral ORF57 are still to be explored. It should be noted that the specific RNA recognition by flexible charged polypeptide regions is not unprecedented, and similar examples have been described in the literature [46]–[49]. The mapping of RNA binding regions using NMR is also fully supported by the biochemical data. Mutations of selected residues (Y81+R82, R88+F89 and W108+R111+V112) to alanine within these regions reduced the efficiency of UV RNA-ORF57^8–120^ cross-linking, confirming their involvement in mediating RNA binding (Fig. 7A). Moreover, earlier we probed the physiological effect of a number of mutations in this region via an ex vivo assay for cytoplasmic accumulation of an HVS ORF47 reporter mRNA, which reflects the ability of full-length ORF57 (bearing site-specific mutations) to form an export competent ribonucleoprotein particle [30]. Mutations within R-b helix of W108A, R111A, V112A, R119A and R120A and their combinations all substantially reduced the efficiency of the mRNA cytoplasmic accumulation, which was previously interpreted as a confirmation of the functional significance of ORF57 – ALYREF interaction for ORF57-mediated nuclear export of viral mRNA [30]. In view of the new experimental data, the same R-b-helix region is also directly involved in viral mRNA binding, meaning that the physiological effect of these mutations cannot be solely attributed to blocking ORF57 – ALYREF interactions, but these mutations will likely affect the whole process of how viral mRNA is recognized and introduced to ALYREF. Additionally, mutations R79A+V80A and R94A+I95A in the flanking region also reduced noticeably the cytoplasmic accumulation [30], which now can be explained by the involvement of this region in mRNA binding. These previously obtained physiological assay data obtained with full-length proteins thus corroborate the functional importance of the molecular regions characterized here in detail using shorter molecular fragments. Future in vivo studies using all these ORF57 mutations introduced in live HVS, and monitoring their effect on the process of cell infection and time-dependent localization of interacting components, are expected to clarify further the order of binding events mediated by the R-b-helix and flanking RNA-binding regions in the context of live cell. The mutations most detrimental to HVS are expected to involve residues situated on the R-b helix which participate in both ALYREF and viral RNA binding. The results presented here provide a map for further extensive mutational analysis, and a framework for its functional interpretation in vivo.

### Structure of ALYREF- ORF57 interaction interface

The ALYREF-ORF57 structure determined here shares some similarities with the ALYREF-ICP27 structure [30] (Fig. 3) as both viral peptides make similar contacts with a patch on ALYREF's surface. There are however clear differences between the ALYREF-ICP27 and ALYREF-ORF57 complexes, not discovered in the earlier signal perturbation mapping analysis [30]. The ORF57 fragment is helical and mainly contacts the looped side of ALYREF, whereas ICP27 has an extended conformation and stretches along the groove formed by α-helices of ALYREF RRM (Fig. 3). As a result, the ALYREF-ORF57 complex is superficially more similar to U2AF homology motif (UHM) recognition [50] of Trp containing peptides (Fig. 3D), although ALYREF lacks the signature Arg-X-Phe UHM interacting motif [51]. It is therefore apparent that variations in sequences and local structures of viral adaptor proteins can achieve similar binding with the promiscuous RRM domain of ALYREF [52]. This may explain the lack of an obvious conserved “ALYREF-binding motif” in other herpes viral adaptor proteins, such as KSHV ORF57 or EBV EB2 [17], [18], [53].

We previously suggested a recognition triad for ALYREF, namely W105, R107, L108 in ICP27 and W108, R111, V112 in ORF57 [30]. The functional role of this triad in ICP27-ALYREF binding *in vivo*, and for efficiency of viral mRNA export and HSV-1 production was also studied in detail recently [54]. In light of the new structural data presented here, the importance of R113^ORF57^ should also be emphasized as it plays a similar role to that of R107^ICP27^ [30]. The quantitative differences in mutational effects of the triad residues W105A^ICP27^/W108A^ORF57^ and L108A^ICP27^/V112A^ORF57^ on binding with ALYREF [30] can now be explained by the subtle differences in their structural context. The structure of ORF57-ALYREF complex also provides an explanation for the weak affinity of a short ORF57 aa105–115 peptide and ORF57^8–120^ double mutant R119A+R120A [30], as both constructs are likely to disrupt helix formation and increase the entropic cost of binding.

### Cooperative and flexible nature of the ORF57-RNA-ALYREF complex assembly

Using the combination of traditional atomic-resolution structure determination and novel saturation-transfer experiments we were able to follow the process of RNA transfer from ORF57 to ALYREF in a site-specific manner, and suggest a model of how the ternary complex is assembled (Fig. 8). It is interesting that the signal intensity of the N-terminal region aa22–48 of ALYREF does not reduce significantly upon RNA binding in the presence of ORF57, suggesting that this interaction is relatively transient. Overall, we conclude that ORF57 simply bridges the interaction between viral RNA and cellular ALYREF, cooperatively enhancing the formation of the ternary complex, without allosterically remodeling ALYREF. The overall cooperativity of the ternary complex formation is demonstrated by the quantitative *K_d_* measurements for the different complexes within the thermodynamic cycle (Fig. 6B). The cooperativity of interactions explains the partial RNase sensitivity of the ALYREF-ORF57 complex reported previously [21]. The same cooperativity may also explain why the presence of ORF57 in the nucleus of an infected cell does not cause indiscriminate export of non-viral mRNAs which lack the specific viral sequence motif. The estimates using NMR and fluorescence experiments showed that the apparent *K*_d_ of binding of specific RNA oligonucleotide to equimolar ALYREF^1–155^ – ORF57^8–120^ complex (1.55 µM) is lower than to ALYREF^1–155^ (>50 µM) or ORF57^8–120^ (7.57 µM) individually. The inferred *K*_d_ of ALYREF binding to ORF57-RNA complex (Fig. 6B) is 0.52 µM, two orders of magnitude tighter than for ALYREF-RNA binding. These estimates support that the ternary complex overall is stabilized cooperatively, despite the presence of local competition between RNA and ALYREF for ORF57 R-b helix region aa106–120.

It is notable that the values of NMR signal shifts within the flexible regions were not a good indicator of the formation of transient complexes with RNA. It is likely to be due to a substantial fluidity of the complex leading to extensive chemical shift averaging over the conformational ensemble; however the saturation transfer from RNA protons to protein amides served as a more reliable indicator of local binding. The ternary complex formed here may present a good example of fuzzy complexes, the existence of which was postulated recently [55], [56]; more specifically, it would fit the “flanking” model, where the short R-b helix acts as a clamp forming more rigid part of the complex interface, whereas transient interactions within flexible flanking regions contribute to the overall stability of the complex. Such a mode of recognition, using fairly short linear motifs located within flexible regions of viral proteins, is expected to provide evolutionary advantages for quick adaptability of viruses [56]–[59], and may fit well with a necessity to bind and remodel NXF1, and dismantle the RNA-ALYREF-ORF57 complex at the next step of the viral mRNA export pathway.

### Implications for viral mRNA export

The protein constructs used here comprise the main binding elements for the assembly of the specific ORF57-RNA-ALYREF ternary complex, which is responsible for introducing the herpesviral mRNA to the cellular export factor ALYREF. However, both native ALYREF and ORF57 contain additional regions which may contribute to the binding of longer viral mRNA molecules, adding to the overall cooperativity of this assembly, and strengthening it further. The viral mRNA transcripts, which are much longer than oligos used in the current study, would provide additional contact points for RNA-binding regions within the C-terminal regions of both full-length ALYREF [7], [8] and ORF57 [23]. Therefore, the quantitative measurements of binding reported here for the essential core of the ternary complex provide only a lower affinity estimate for the full-length complex. We speculate that once the ternary ORF57-mRNA-ALYREF complex encounters NXF1-p15, viral mRNA will be displaced from the N-terminus of ALYREF where NXF1 binds in its place [5], [10]. Viral mRNA at that moment will still be retained by ALYREF's RRM domain together with ORF57, presenting it to NXF1. Binding of ALYREF switches NXF1 into a high-affinity RNA-binding mode [5], [6], forcing it to accept the foreign viral mRNA and commit it to export to the cytoplasm. As indicated earlier, the adaptor proteins homologous to HVS ORF57 (ICP27 in HSV-1, ORF57 in KSHV) are expressed by all herpesviridiae. The location of RNA binding sites within these proteins is poorly conserved, and the exact location of binding sites with cellular adaptors such as ALYREF [17] and UIF [19] often is unknown or not evident due to the lack of recognizable sequence motifs responsible for such binding. Even when superficial similarity exists, as in the case of ALYREF recognition triad residues suggested earlier for HSV-1 ICP27 and HVS ORF57 [30], here we showed that in fact the structural details of the molecular recognition are significantly different, despite binding occurring in the same cleft on the surface of the RRM of ALYREF. This finding means that it is probably too early for modeling and predictions to be used to discover the binding interfaces between RNA and viral and cellular proteins, and detailed experimental structural studies need to be continued for this molecular pathogen-host system, with different herpesviruses using diverse strategies for molecular recognition to achieve a similar functional outcome, such as viral mRNA export. Continuation of similar studies for signature viral adaptors from more medically relevant herpesviruses, such as HSV-1 or KSHV involved in cancer, may possibly identify new drug targets for novel treatments.

This work for the first time suggests a detailed mechanism for the assembly of the key ternary RNA-ORF57-ALYREF complex leading to herpesvirus highjacking the host nuclear export pathway. We show the importance of partially-overlapping multifunctional binding sites and combination of competitive and cooperative binding events as a likely mechanism for the orderly assembly and disassembly of mRNA nuclear export complexes and molecular transfer, adding to the emerging knowledge in this area [35], [36], [60], [61].

## Materials and Methods

### Protein expression and purification

All proteins were expressed in *E. coli BL21-RP* cells (*Novagen*). For NMR studies, murine ALYREF (also called REF2-I) isoform constructs aa1–155 (ALYREF^1–155^), aa54–155 (ALYREF^54–155^), as well as ORF57^56–140^ and ORF57^8–120^, were produced as described previously [30]. ALYREF protein constructs are identical to protein REF used in [30]; only name was changed due recent gene naming conventions [31]. ORF57^103–120^ peptide was produced as a GST-fusion construct as described for ICP27^103–138^ [30]. Post gel filtration, all samples were buffer exchanged using an Amicon ultrafiltration cell to the NMR buffer (20 mM phosphate pH 6.2, 50 mM NaCl, 50 mM each of L-Arg, L-Glu and β-mercaptoethanol, 10 mM EDTA); L-Arg and L-Glu were added to reduce protein aggregation and improve sample stability [62]. Unlabelled synthetic peptide ORF57^103–120^ was obtained from Peptide Protein Research Ltd (UK). For UV cross-linking, hexa-histidine ORF57^8–120^, hexa-histidine GB1 and GST-ALYREF fusions were purified by affinity chromatography and dialyzed against RB100 buffer (25 mM HEPES pH 7.5, 100 mM KOAc, 10 mM MgCl_2_, 1 mM DTT, 0.05% Triton, 10% Glycerol).

### NMR experiments

All experiments were carried out at 30°C on Bruker DRX600, DRX700 and Varian Inova 800 MHz spectrometers equipped with cryoprobes, and a Bruker DRX800 with a room temperature probe. Standard triple-resonance experiments were used to assign spectra: ORF57^103–120^ in free form and with a 3-fold excess of unlabelled ALYREF^54–155^ added, ORF57^56–140^ in the free form, and ALYREF^54–155^ with a 3-fold excess of ORF57^103–120^ synthetic peptide added. ALYREF^54–155^, ALYREF^1–155^ and ORF57^8–120^ were assigned previously [10], [30]. Spectra were processed using NMRpipe [63] and Topspin 2.1 (*Bruker*) and analyzed using Sparky (University of California). Distance restraints obtained from 3D ^15^N- and ^13^C- edited NOESY-HSQC experiments (τ_m_ 130 ms) and dihedral restraints from TALOS+ [42] were used in structure calculations by CYANA [64]. Additionally, intermolecular contacts were unambiguously identified using ^13^C-edited, ^12^C-filtered NOESY-HSQC (τ_m_ 150 ms) spectra acquired on a Varian Inova 800 MHz spectrometer. In this experiment only NOE crosspeaks between ^1^H-^13^C moieties of ^13^C,^15^N-labelled ALYREF and ^1^H(^12^C) of unlabelled ORF57 were observed [65], [66]. A final ensemble contained 20 structures with lowest target function values. Images were generated using Pymol (*DeLano Scientific*). Chemical shift assignments were submitted to the BioMagResBank for free ORF57^103–120^ (bmr17664), free ORF57^56–140^ (bmr17663) and the ALYREF-ORF57 complex (bmr17693). Structure coordinates and experimental constraints for ALYREF-ORF57 complex were deposited into the Protein Data Bank (2yka). Ramachandran plot statistics for residues in most favored regions, additional allowed regions, generously allowed regions, disallowed regions calculated for structured ALYREF^74–152^+ORF57^106–120^ are: 79.8%, 20.2%, 0%, 0%.

IDIS-TROSY spectra [38] were acquired using 1∶1 mixtures of 0.4 mM ^13^C,^15^N-labelled ORF57^8–120^ and ^15^N-labelled ALYREF^1–155^ or ALYREF^54–155^, followed by additions of RNA. RNA oligonucleotides were obtained from Sigma. Two oligos contained the ORF57-specific motif [32] GAAGAGG (7merS) and CAGUCGCGAAGAGG (14merS), and two were non-specific CAGUCGC (7merN) and CAGUCGCAUAGUGCA (15merN; this oligo is identical to that used previously [10]). Irradiation of resolved RNA signals [39] in cross saturation transfer (ST) [37] versions of standard Bruker-library HSQC, TROSY and IDIS-TROSY [38] was achieved by using a selective Gaussian pulse train (lasting 0.7 s in total) using a series of 8.5 ms 180 degree pulses (B_1_ = 60 Hz). The saturation pulse train was tagged at the end of relaxation delay of 2.3 s, immediately prior to the first hard proton pulse. RNA peaks were selectively irradiated by centering the pulse train at 5.85, 5.75 or 12.00 ppm frequencies, with off-resonance irradiation at 21 ppm. Ratios of amide signal intensities in equivalent spectra, obtained with saturation at two different frequencies *freq1* and *freq2* (as indicated) were obtained. Residues were highlighted as close to RNA in space if the ratio of signal intensities I*^freq1^*/I*^freq2^* differed significantly from unity (by more than three standard deviations (SD), calculated from the I*^freq1^*/I*^freq2^* variability observed within non-interacting regions).

### UV cross-linking of RNA with ALYREF and ORF57

RNA oligonucleotides were end-labelled with [γ^32^P]-ATP using Polynucleotide Kinase (*Fermentas*). UV cross-linking with proteins was performed as previously described [10]. For the *in vitro* reconstitution assay, 10 or 100 µg ORF57^8–120^ was incubated with 5 µg radiolabelled and cold RNA (7merS or 14merS) at room temperature for 10 minutes. The mixture was added to 20 µg of GST-tagged full length ALYREF (aa1–218) immobilized onto Glutathione-coated beads (*GE Healthcare*) in RB100 buffer. Beads were washed and complexes were eluted in native conditions (50 mM TRIS pH 7.5, 100 mM NaCl, 40 mM reduced glutathione) before being subjected to UV-irradiation or not. Proteins were resolved on 15% SDS-PAGE stained with Coomassie blue and analyzed by PhosphoImaging.

### Fluorescence experiments

Purified proteins were transferred into buffer F (20 mM phosphate pH 6.2, 50 mM NaCl, 50 mM L-Arg+L-Glu, 5 mM EDTA, 1 mM TCEP) by 3 overnight dialysis steps and then concentrations determined by UV absorption (280 nm). Measurements were carried out on a Varian Cary Eclipse fluorimeter, with excitation at 280 nm and emission monitored over 290–600 nm at a scan rate of 120 nm/min. Titrations were carried out with at least 1 min of equilibration time after each addition. ORF57^8–120^-RNA (OR) titrations were performed using 13 µM ORF57, titrations of 1∶1 ORF57-ALYREF (OA) with RNA used an initial protein concentrations of 2.5 and 10 µM. Blue shift in emission maximum was caused by protein-protein (OA and OAR) complex formation and was quantified as a change in barycentric mean values 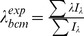 (“centre of mass” of the peak), where *λ* is emission wavelength (320–390 nm) and *I_λ_* is fluorescence intensity at this wavelength. Binding of RNA to ORF57 (in OR and OAR complexes) was quantified by measuring a decrease in integral fluorescent intensity 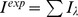. Apparent macroscopic dissociation constants *K_d_* for binary complexes were obtained by non-linear regression fit of 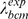 and 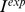 dependences on the total concentration of added component, using either a standard quadratic equation, or DynaFit software (BioKin Ltd) [43] which produced the same results. Value of apparent macroscopic *K_d_* for ternary complex formation was obtained by titrating RNA (14merS) to 10 µM 1∶1 ALYREF-ORF57 mixture, followed by simultaneous fitting of the associated changes in 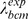 and 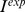 to the non-redundant three-equation equilibrium model (Fig. 6A) using DynaFit [43]. Changes in normalized fluorescence parameters, caused by the increase in [OA] or/and [R], were related with concentrations as 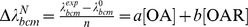, and 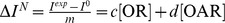, with the values of the response coefficients *a*, *b*, *c* and *d* and normalization factors *n* and *m* obtained during the nonlinear fit (so that *a* = 1≈*b* and *c* = 1≈*d*, and all concentration expressed in µM units). Further simulations of equilibrium reactions within different binding models were conducted using COPASI software [67].

## Supporting Information

Figure S1 **NMR mapping of RNA interactions with ORF57, ALYREF and the ALYREF-ORF57 complex.** (A) Chemical shift changes in ORF57^8–120^ induced by addition of RNA oligos: blue diamonds - 7merS (ORF57:RNA ratio of 1∶1), blue crosses - 7merS (1∶0.5), green open triangles - 7merN (1∶2) and green closed triangles - 15merS (1∶2). Blue diamonds positioned at the top mark residues with signals broadened beyond detection. (B) ^15^N{^1^H}-NOEs do not change significantly in response to addition of non-specific RNA: blue diamonds are for free ORF57^8–120^, blue crosses - ORF57^8–120^:7merN (1∶2), red squares - free ORF57^56–140^, and red dashes - ORF57^56–140^:7merN (1∶2). (C) Overlay of ^1^H-^15^N HSQC spectra of ORF57^103–120^ in the absence of RNA (red) and with three-fold excess of RNA 7merS (green) added. Sidechain amide signals are labeled with asterisks. (D) Scheme illustrating the original principle of the ST-HSQC cross-saturation experiment for determining the binding interfaces between RNA and ^15^N-labeled protein [37], [39]. RNA signals are selectively saturated by radiofrequency (RF) pulses, which causes change in NMR signal intensity of adjacent protein amides (detected in ^1^H-^15^N correlation spectra). (E) Saturation transfer from 7merS (green) and 7merN (red) to ORF57 amides, using ORF57:RNA ratios of 1∶0.5 and 1∶2 respectively, measured by ST-HSQC experiment. (F) Saturation transfer from 14merS RNA to the N-terminal region of ALYREF^1–155^ (ALYREF∶RNA ratio 1∶0.5) measured by ST-HSQC. ST-IDIS-TROSY (G and H) was used to simultaneously detect RNA contacts with ORF57^8–120^ (*i*) and ALYREF^1–155^ (*ii*) when these two differentially-labeled proteins and unlabelled RNA formed a single equimolar complex. ST from 14merS to ORF57^8–120^ and ALYREF^1–155^ in complex are shown for two saturation schemes, with calculated ratios of signal intensities I^5.75^/I^5.85^ (F) and I^12.0^/I^21.0^ (H). Dashed horizontal lines show the levels of three standard deviations from the mean baseline values.
(EPS)

Figure S2 **NMR derived evidence for presence of an α-helix in ORF57.** ORF57 region aa108–118 is α-helical. (A) Long axis view of helix showing its amphipathic nature. (B) Model constructed using experimental dihedral angles and NOE restrains. (C) Position of the α-helix (named here R-b helix) determined from backbone dihedral angle values using TALOS+ (Shen, Y., et al. 2009, *J Biomol NMR* **44**, 213–223) in three ORF57 constructs with different domain boundaries.
(TIF)

Figure S3 **Overview of spectral data for ALYREF^54–155^-ORF57^103–120^ complex.** ^1^H-^15^N-HSQC spectra of (A) ALYREF^54–155^ free (red) and bound to HVS ORF57^103–120^ (blue) or bound to HSV-1 ICP27^103–138^ (pale green), and (B) ORF57 free (red) and bound to ALYREF^54–155^ (blue). Both viral peptides cause perturbations within same ALYREF residues showing that they bind to the same site, but the direction of signal movements mostly differ, suggesting some differences between the interactions. Sidechain amide signals are labeled * and signals from N- or C-terminal tag sequences are labeled x. NMR derived parameters obtained for ALYREF-ORF57 and ALYREF-ICP27 interactions are summarized for (C) ORF57^103–120^ and (D) ALYREF^54–155^. Residues with large and moderate reductions of mobility upon binding, as evidenced by the increase in ^15^N[^1^H] NOE, are highlighted by solid and broken bars mark, respectively. Similarly, large and moderate changes in chemical shifts (δCS) of amide signals are shown as solid and broken bars, respectively. Red or orange bars mark residues forming direct inter-molecular NOE contacts in ALYREF-ORF57 or ICP27 complexes, respectively. The position of secondary structure elements (α-sheets, β-helices and loops) is shown. Data for ORF57^8–120^ and ICP27^103–138^ marked with asterisks was previously released (Tunnicliffe, R. B., et al. 2011, *PLoS Pathog* **7**, e1001244.) and is included here for comparison.
(EPS)

Figure S4 **NOE-derived distance constraints used in the structure calculation of the ALYREF^54–155^ : ORF57^103–120^ complex.** The position of short and medium range NOE connectivities are shown in (A) for ALYREF and (B) for ORF57. The sequences colored blue highlight tags introduced in cloning. (C) The distribution of all NOEs on a per residue basis. White, light grey, dark grey and black shading of bars indicates the number of meaningful intra-residue, sequential (i+1), medium (2≤i≤4) and long (5≤i) range constraints. Two samples were used for structure determination of the ORF57^103–120^: ALYREF^54–155^ complex, these contained one protein ^13^C,^15^N uniformly labeled at 1 mM plus the binding partner in unlabelled form at 3 mM. Over-titration of the labeled component was necessary to observe the signals otherwise broadened in the equimolar complex. (D) Intermolecular NOE restraints used in structure calculations are shown schematically between the individual residues of ALYREF and ORF57. Positions of two α-helices of ALYREF are marked. Each line corresponds to a non-redundant NOE restraint. Dark green continuous lines represent NOEs obtained unambiguously from ^13^C edited, ^12^C-filtered NOESY-HSQC spectra. Additional NOEs represented by light green dashed were obtained from more sensitive standard 3D ^13^C-resolved NOESY-HSQC spectra. (E) Example sections of 3D ^13^C edited, ^12^C-filtered NOESY-HSQC spectra showing intermolecular NOEs. Positive signals are colored green and negative red. ORF57 ^1^H signal assignments are shown in blue and ALYREF ^1^H assignments shown as vertical orange dashed lines. The experiment selects NOE cross peaks between ^1^H(^12^C) of unlabelled ORF57^103–120^ and ^1^H-^13^C moieties of [^13^C,^15^N]- ALYREF^54–155^, therefore providing exclusively inter-molecular restraints.
(EPS)

Figure S5 **^1^H-^15^N correlation spectra of ALYREF^1–155^ (** ***i*** **) and ORF57^8–120^ (** ***ii*** **) in different binding states illustrate that the presence of ALYREF partially shields ORF57 from binding specific RNA 14merS.** Where indicated with arrows, the spectra of differentially-labeled proteins were acquired simultaneously in the same sample using IDIS-TROSY experiment. (A) TROSY spectra of proteins in free form (red) are overlaid with IDIS-TROSY of a 1∶1 complex (blue). The signals from residues at the protein-protein interface are exchange broadened, but signal shifts from other residues nearby are characteristic to complex formation. (B) An equimolar amount of HVS-specific RNA oligo 14merS was added to this ALYREF:ORF57 complex (grey), which induced signal changes mostly in ALYREF and to small extent in ORF57. (C) For comparison, addition of the same RNA oligo to ALYREF in the absence of ORF57 causes identifiable signal shifts on ALYREF, as revealed by comparison of HSQC spectra. Addition of the same RNA oligo to ORF57 in the absence of ALYREF causes very extensive peak broadening in the whole C-terminal half of ORF57^8–120^. The examples of IDIS-TROSY spectra shown in (A) and (B) illustrate the ability to monitor in both proteins the individual signal changes caused by inter-protein complex formation, and further changes on both proteins caused by the addition of RNA.
(EPS)

Figure S6 **The local ORF57-ALYREF interaction is stronger than the ORF57-RNA.** Same type of experiments are presented as shown on Figure S5, but using ALYREF^54–155^ (*i*) (which lacks the N-terminal RNA-binding region), ORF57^8–120^ (*ii*) and a shorter specific RNA 7merS. (A) TROSY spectra of protein in free form (red) are overlaid with IDIS-TROSY of a 1∶1 complex (blue); signals from residues at the protein-protein interface are exchange broadened, while movements of signals from residues close to the interface are characteristic to protein-protein complex formation. (B) An equimolar amount of HVS-specific RNA oligo 7merS was added to this complex (grey), which induced very limited signal changes in ALYREF and even less in ORF57. (*C*) For comparison, addition of the same RNA oligo to ORF57 in the absence of ALYREF causes very extensive peak broadening in the whole C-terminal half of ORF57^8–120^.
(EPS)

Figure S7
**Estimates of binding affinities using fluorescence.** ORF57^8–120^ (13 µM) was titrated with RNA oligos 14merS (A) and 7merN (B), and change in integral fluorescence emission intensity *ΔI* was monitored. The values of *K*_d_ shown were obtained using DynaFit software (BioKin Ltd) [43]. The satisfactory fit to the experimental data could be achieved only assuming 1∶1 binding stoichiometry. (C) Formation of protein-protein complex (by increasing concentrations of ORF57^8–120^: ALYREF^1–155^, [O,A]) causes blue shift of fluorescence emission peak, as measured by a change in λ_bcm_. The upper panel shows fluorescence spectra normalized by protein concentration, with concentration for each sample in µM units marked on the right. The bottom panel shows non-linear best-fit of 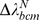 to the standard quadratic equation; the estimated apparent *K*_d_ for ORF57-ALYREF binding is 2.56 µM. (D) Addition of specific RNA oligo to equimolar mixture of ORF57^8–120^: ALYREF^1–155^ (OA) first further enhances blue shift of fluorescence which is consistent with the ternary complex formation, however overtitrating this complex with excess RNA (above 5-fold) causes fluorescence maximum shift in the opposite direction, consistent with dissociation of protein-protein complex formed earlier. The inset illustrates typical behavior of the fluorescence signal in response to addition of RNA to 2.5 µM protein-protein complex. Experimentally measured values for 10 and 2.5 µM complexes are represented with diamonds and triangles, respectively. The solid lines represent typical theoretical binding curves calculated using equilibrium models shown on Fig. 6B,C and COPASI software [67]. These models adequately fit the experimental data when concentration of RNA is close to stoichiometric, but fail to describe correctly the competitive binding of RNA to ORF57 and ALYREF separately, when RNA is in large excess, leading to ternary complex dissociation. More sophisticated binding models, with more parameters included, and more measurable observed experimentally, would be needed in the future to describe the binding behavior of this complex system in the wider range of component concentrations.(TIF)
