# Supplementary figures and images for: Competitive and Cooperative Interactions Mediate RNA Transfer from Herpesvirus Saimiri ORF57 to the Mammalian Export Adaptor ALYREF
Source: PLoS Pathog. 2014 Feb 13;10(2):e1003907. doi: 10.1371/journal.ppat.1003907 (PMC3923783; doi:10.1371/journal.ppat.1003907)

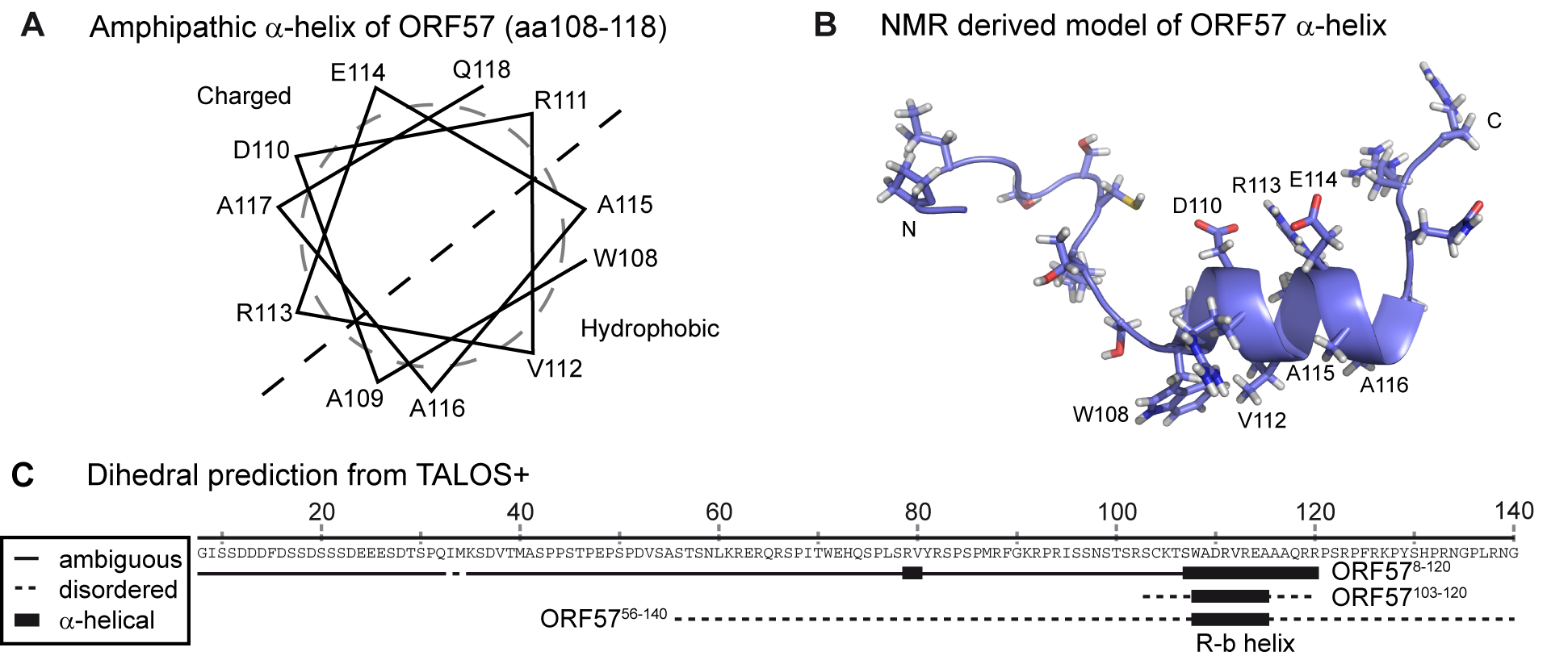

Supplement: Figure S2 — NMR derived evidence for presence of an α-helix in ORF57. ORF57 region aa108–118 is α-helical. (A) Long axis view of helix showing its amphipathic nature. (B) Model constructed using experimental dihedral angles and NOE restrains. (C) Position of the α-helix (named here R-b helix) determined from backbone dihedral angle values using TALOS+ (Shen, Y., et al. 2009, J Biomol NMR 44, 213–223) in three ORF57 constructs with different domain boundaries. (TIF) [file ppat.1003907.s002.tif]

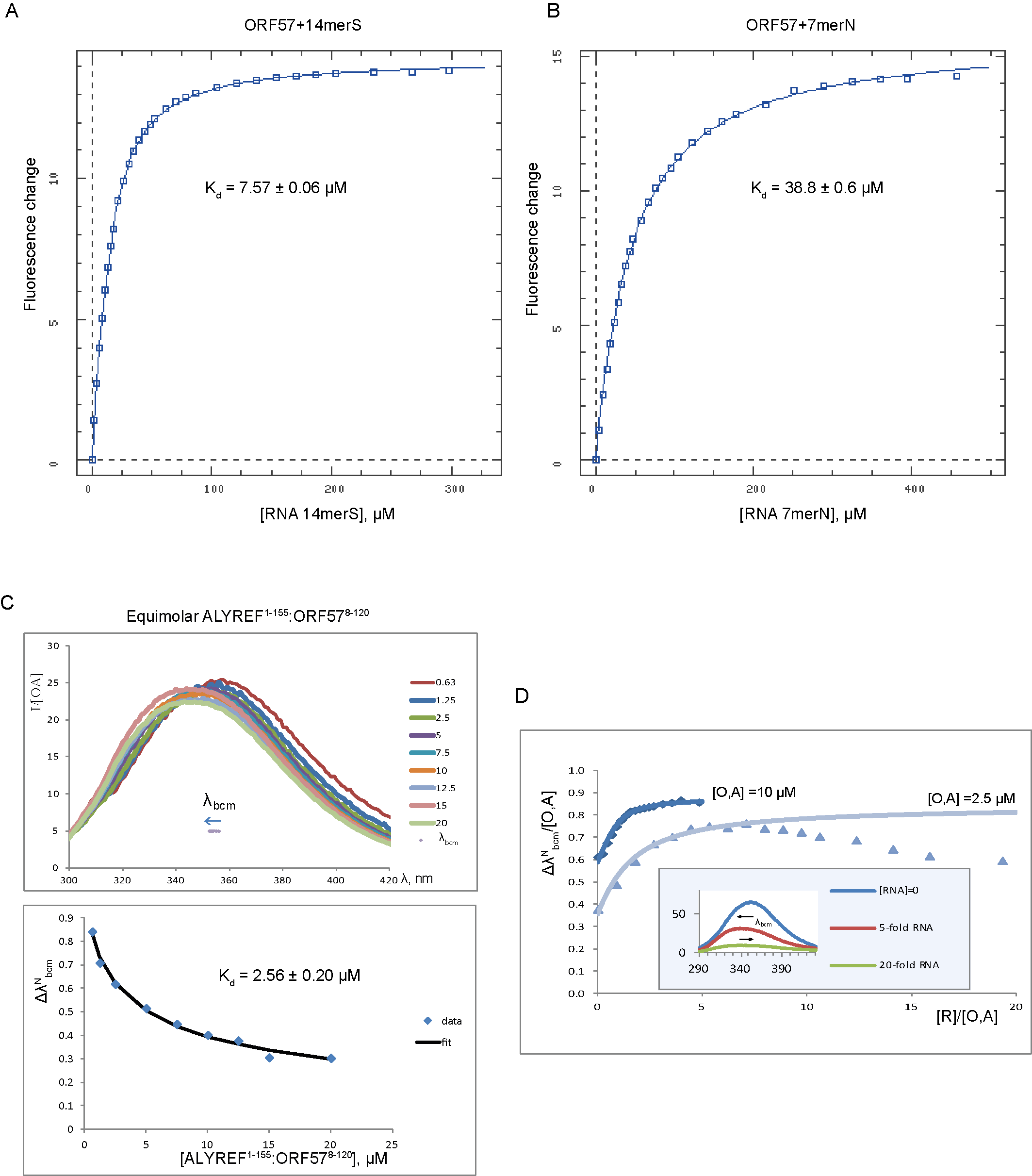

Supplement: Figure S7 — Estimates of binding affinities using fluorescence. ORF578–120 (13 µM) was titrated with RNA oligos 14merS (A) and 7merN (B), and change in integral fluorescence emission intensity ΔI was monitored. The values of K d shown were obtained using DynaFit software (BioKin Ltd) [43]. The satisfactory fit to the experimental data could be achieved only assuming 1∶1 binding stoichiometry. (C) Formation of protein-protein complex (by increasing concentrations of ORF578–120: ALYREF1–155, [O,A]) causes blue shift of fluorescence emission peak, as measured by a change in λbcm. The upper panel shows fluorescence spectra normalized by protein concentration, with concentration for each sample in µM units marked on the right. The bottom panel shows non-linear best-fit of to the standard quadratic equation; the estimated apparent K d for ORF57-ALYREF binding is 2.56 µM. (D) Addition of specific RNA oligo to equimolar mixture of ORF578–120: ALYREF1–155 (OA) first further enhances blue shift of fluorescence which is consistent with the ternary complex formation, however overtitrating this complex with excess RNA (above 5-fold) causes fluorescence maximum shift in the opposite direction, consistent with dissociation of protein-protein complex formed earlier. The inset illustrates typical behavior of the fluorescence signal in response to addition of RNA to 2.5 µM protein-protein complex. Experimentally measured values for 10 and 2.5 µM complexes are represented with diamonds and triangles, respectively. The solid lines represent typical theoretical binding curves calculated using equilibrium models shown on Fig. 6B,C and COPASI software [67]. These models adequately fit the experimental data when concentration of RNA is close to stoichiometric, but fail to describe correctly the competitive binding of RNA to ORF57 and ALYREF separately, when RNA is in large excess, leading to ternary complex dissociation. More sophisticated binding models, with more parameters included, and more measur [file ppat.1003907.s007.tif]
